# Supplementary material for: Myosin Binding Protein-C Forms Amyloid-Like Aggregates In Vitro
Source: Int J Mol Sci. 2021 Jan 13;22(2):731. doi: 10.3390/ijms22020731 (PMC7828380; doi:10.3390/ijms22020731)
Supplement: Supplementary file 1 [file ijms-22-00731-s001.zip › ijms-1057004 SI-done/ijms-1057004-supplementary.docx]

Myosin Binding Protein-C Forms Amyloid-Like Aggregates In Vitro

Liya G. Bobyleva ^1^, Sergey A. Shumeyko ^1^, Elmira I. Yakupova ^1^, Alexey K. Surin ^2,3,4^, Oxana V. Galzitskaya ^1,2^, Hiroshi Kihara ^5^, Alexander A. Timchenko ^6^, Maria A. Timchenko ^7^, Nikita V. Penkov ^8^, Alexey D. Nikulin ^9^, Mariya Yu. Suvorina ^2^, Nikolay V. Molochkov ^10^, Mikhail Yu. Lobanov ^2^, Roman S. Fadeev ^11^, Ivan M. Vikhlyantsev ^1,^* and Alexander G. Bobylev ^1,^*

^1^ Laboratory of the Structure and Functions of Muscle Proteins, Institute of Theoretical and Experimental Biophysics, Russian Academy of Sciences, 142290 Pushchino, Russia;
[liamar@rambler.ru](mailto:liamar@rambler.ru) (L.G.B.); [shumik92@gmail.com](mailto:shumik92@gmail.com) (S.A.S.); [yakupova.mira@mail.ru](mailto:yakupova.mira@mail.ru) (E.I.Y.);
[ogalzit@vega.protres.ru](mailto:ogalzit@vega.protres.ru) (O.V.G.)

^2^ Laboratory of Bioinformatics and Proteomics, Institute of Protein Research, Russian Academy of Sciences, 142290 Pushchino, Russia; [alan@vega.protres.ru](mailto:alan@vega.protres.ru) (A.K.S.); [marrruko@yandex.ru](mailto:marrruko@yandex.ru) (M.Y.S.); [m.u.lobanov@mail.ru](mailto:m.u.lobanov@mail.ru) (M.Y.L.)

^3^ Biological Testing Laboratory, Branch of the Shemyakin–Ovchinnikov Institute of Bioorganic Chemistry, Russian Academy of Sciences, 142290 Pushchino, Russia

^4^ Laboratory of the Biochemistry of Pathogenic Microorganisms, State Research Centre for Applied Microbiology and Biotechnology, Obolensk, 142279 Serpukhov District, Russia

^5^ Department of Early Childhood Education, Himeji-Hinomoto College, 890 Koro, Kodera-cho, Himeji 679-2151, Japan; [kiharah1234@gmail.com](mailto:kiharah1234@gmail.com)

^6^ Group of Experimental Research and Engineering of Oligomeric Structures, Institute of Protein Research, Russian Academy of Sciences, 142290 Pushchino, Russia; [atim@vega.protres.ru](mailto:atim@vega.protres.ru)

^7^ Laboratory of Applied Enzymology, FRC PSCBR, Russian Academy of Sciences, 142290 Pushchino, Russia; [maria_timchenko@mail.ru](mailto:maria_timchenko@mail.ru)

^8^ Laboratory of the Methods of Optical Spectral Analysis, Institute of Cell Biophysics, Russian Academy of Sciences, FRC PSCBR RAS, 142290 Pushchino, Russia; [nvpenkov@rambler.ru](mailto:nvpenkov@rambler.ru)

^9^ Laboratory for Structural Studies of the Translational Apparatus, Institute of Protein Research, Russian Academy of Sciences, 142290 Pushchino, Russia; [nikulin@vega.protres.ru](mailto:nikulin@vega.protres.ru)

^10^ Laboratory of NMR Investigations of Biosystems, Institute of Theoretical and Experimental Biophysics, Russian Academy of Sciences, 142290 Pushchino, Russia; [nmolochkov@gmail.com](mailto:nmolochkov@gmail.com)

^11^ Laboratory of Pharmacological Regulation of Cell Resistance, Institute of Theoretical and Experimental Biophysics, Russian Academy of Sciences, 142290 Pushchino, Russia; [fadeevrs@gmail.com](mailto:fadeevrs@gmail.com)

***** Correspondence: (I.M.V.); [bobylev1982@gmail.com](mailto:bobylev1982@gmail.com) (A.G.B.)

**Supplementary Materials and Figures**

| **Protein Group** | **Protein ID** | **Accession** | **Score (%)** | **-10lgP** | **Coverage**  **(%)** | **Peptides** | **Unique** | **Avg. Mass** | | **Description** |
| --- | --- | --- | --- | --- | --- | --- | --- | --- | --- | --- |
| **1** | 23228 | MYBPC2 | 99.2 | 696.69 | 85 | 503 | 499 | 127206 | | sp\|MYBPC2 |
| **2** | 23231 | MYBPC1 | 98.0 | 108.49 | 7 | 10 | 6 | 119153 | | sp\|MYBPC1 |
|  |  |  |  |  |  |  |  |  |  |  |

**Supplementary Table S1. Mass spectrometric data of a purified MyBP-C preparation**

**Supplementary Table S2. List of peptides determined by HPLC–MS in purified MyBP-C hydrolysate**

| **Peptide** | **Uniq** | **Score (%)** | **-10lgP** | **Mass** | **Length** | **ppm** | **m/z** | **z** | **RT** | **PTM** |
| --- | --- | --- | --- | --- | --- | --- | --- | --- | --- | --- |
| K.GKDAPKEEPPKEAAAEPPK.E | Y | 100.0 | 144.36 | 1988.0214 | 19 | -0.7 | 498.0123 | 4 | 33.51 |  |
| F.LKKPDSVSVETGKDAVIM(+15.99)AK.L | Y | 100.0 | 143.42 | 2131.1558 | 20 | 0.5 | 533.7965 | 4 | 33.72 | Oxidation (M) |
| L.SAKLNFLEIKVEYVPKQEPPKIH.L | Y | 100.0 | 137.14 | 2706.5107 | 23 | 0.5 | 542.3097 | 5 | 105.23 |  |
| F.LKKPDSVSVETGKDAVIM.A | Y | 100.0 | 136.37 | 1916.0288 | 18 | -0.3 | 639.6833 | 3 | 33.84 |  |
| R.KAPKGKDAPKEEPPKEAAAEPPK.E | Y | 100.0 | 133.71 | 2412.3010 | 23 | -0.3 | 483.4673 | 5 | 33.46 |  |
| R.AGDAKPDGAGELDFSGL.L | Y | 100.0 | 132.14 | 1618.7474 | 17 | -0.8 | 810.3803 | 2 | 105.85 |  |
| F.KRAGDAKPDGAGELDFSGLLKKR.E | Y | 100.0 | 129.98 | 2428.3186 | 23 | 1.2 | 486.6716 | 5 | 93.15 |  |
| R.AGDAKPDGAGELDFSGLLKKREVVEEEKKK.K | Y | 100.0 | 129.48 | 3242.7146 | 30 | -0.7 | 649.5497 | 5 | 95.39 |  |
| F.LKKPDSVSVETGKDAVIM(+15.99).A | Y | 100.0 | 129.46 | 1932.0237 | 18 | -1.7 | 645.0141 | 3 | 33.75 | Oxidation (M) |
| R.AGDAKPDGAGELDFSGLLKKR.E | Y | 100.0 | 126.86 | 2144.1226 | 21 | 0.1 | 715.7148 | 3 | 100.21 |  |
| K.GKDAPKEEPPKEA.A | Y | 100.0 | 125.33 | 1394.7041 | 13 | 0.2 | 698.3595 | 2 | 23.80 |  |
| F.NIDVEAPRQDSAGQ.S | Y | 100.0 | 124.53 | 1498.7012 | 14 | -0.9 | 750.3572 | 2 | 33.74 |  |
| R.AGDAKPDGAGELDFSGLLK.K | Y | 100.0 | 115.11 | 1859.9264 | 19 | -2.5 | 930.9681 | 2 | 37.20 |  |
| K.APKGKDAPKEEPPKEA.A | Y | 99.9 | 111.46 | 1690.8889 | 16 | -0.9 | 846.4510 | 2 | 30.85 |  |
| R.KAPKGKDAPKEEPPKEA.A | Y | 100.0 | 111.30 | 1818.9839 | 17 | -1.2 | 607.3345 | 3 | 22.29 |  |
| R.HLRQTYVR.K | Y | 100.0 | 110.21 | 1071.5938 | 8 | -0.1 | 358.2051 | 3 | 33.43 |  |
| R.Q(-17.03)DSAGQSLESFKR.A | Y | 100.0 | 107.21 | 1434.6738 | 13 | 0.4 | 718.3445 | 2 | 33.86 | Pyro-glu from Q |
| L.SDSPGVSKNTAHIL.K | Y | 100.0 | 106.31 | 1424.7260 | 14 | -0.4 | 713.3700 | 2 | 33.80 |  |
| E.ISDPDLPLKWF.K | Y | 99.9 | 105.49 | 1329.6968 | 11 | 0.1 | 665.8557 | 2 | 130.80 |  |
| K.RAGDAKPDGAGELDFSGLLK.K | Y | 100.0 | 101.82 | 2016.0276 | 20 | 0.0 | 673.0165 | 3 | 105.41 |  |
| R.TSDFDTVFFVR.E | Y | 100.0 | 101.74 | 1332.6350 | 11 | -0.8 | 667.3242 | 2 | 34.17 |  |
| R.KAPKGKDAPKEEPPKEAA.A | Y | 99.9 | 101.67 | 1890.0210 | 18 | 0.0 | 631.0143 | 3 | 28.63 |  |
| L.ESFKRAGDAKPDGAGELDFSGLLKKR.E | Y | 100.0 | 101.36 | 2791.4617 | 26 | 0.2 | 559.2997 | 5 | 96.57 |  |
| E.TGKDAVIM(+15.99)AK.L | Y | 100.0 | 101.26 | 1048.5587 | 10 | 0.0 | 525.2866 | 2 | 22.98 | Oxidation (M) |
| T.SAPQHLTVEDVTDTTTTLK.W | Y | 99.9 | 99.17 | 2056.0325 | 19 | -0.8 | 686.3509 | 3 | 93.93 |  |
| Q.VDRGNKVKLVVEISDPDLPLKWFKN.G | Y | 99.9 | 97.72 | 2908.6174 | 25 | 0.0 | 728.1616 | 4 | 118.06 |  |
| A.GDAKPDGAGELDFSGLLK.K | Y | 99.9 | 97.38 | 1788.8893 | 18 | -0.4 | 597.3035 | 3 | 109.50 |  |
| C.EVSDEKVTGK.W | Y | 99.9 | 96.96 | 1090.5505 | 10 | -0.6 | 546.2822 | 2 | 15.78 |  |
| L.VIDDVRPEDEGDYT.F | Y | 99.9 | 96.86 | 1621.7107 | 14 | 0.1 | 811.8627 | 2 | 36.71 |  |
| R.EVVEEEKKKKKDDDDLGIPPEIW.E | Y | 99.9 | 95.60 | 2738.4014 | 23 | -3.1 | 1370.2037 | 2 | 38.33 |  |
| F.KRAGDAKPDGAGELDFSGLLK.K | Y | 99.9 | 95.55 | 2144.1226 | 21 | -1.0 | 537.0374 | 4 | 33.96 |  |
| M.LRVVDVPDPPEAV.R | Y | 99.9 | 94.85 | 1404.7612 | 13 | 0.8 | 703.3884 | 2 | 107.73 |  |
| L.RVVDVPDPPEAV.R | Y | 99.9 | 93.85 | 1291.6771 | 12 | -0.4 | 646.8456 | 2 | 91.97 |  |
| Y.SEVNLEDRGHYQ.V | Y | 99.9 | 93.85 | 1445.6534 | 12 | -1.6 | 723.8328 | 2 | 34.65 |  |
| K.VEYVPKQEPPKIH.L | Y | 99.9 | 92.42 | 1562.8456 | 13 | -0.8 | 782.4294 | 2 | 37.08 |  |
| E.ISDPDLPLKWFKN.G | Y | 99.9 | 92.31 | 1571.8347 | 13 | 0.5 | 786.9250 | 2 | 113.39 |  |
| R.AGDAKPDGAGELDFSGLLKKREVVEEEKKKK.K | Y | 99.9 | 91.57 | 3370.8096 | 31 | 0.5 | 675.1696 | 5 | 92.97 |  |
| C.SFNIDVEAPRQDSAGQSLESFKR.A | Y | 99.9 | 91.36 | 2580.2568 | 23 | -1.2 | 646.0707 | 4 | 100.40 |  |
| V.VEISDPDLPLKWF.K | Y | 99.9 | 90.96 | 1557.8079 | 13 | 1.2 | 779.9121 | 2 | 132.00 |  |
| N.YRFKKDGKRH.I | Y | 99.9 | 89.60 | 1333.7367 | 10 | -0.7 | 334.4412 | 4 | 32.85 |  |
| R.AGDAKPDGAGELDFS.G | Y | 99.9 | 89.52 | 1448.6418 | 15 | -0.7 | 725.3277 | 2 | 33.92 |  |
| F.LKKPDSVSVETGKDAVIM(+15.99)A.K | Y | 99.9 | 87.74 | 2003.0608 | 19 | 0.3 | 668.6944 | 3 | 81.62 | Oxidation (M) |
| K.YVFENVGKKRIL.T | Y | 99.9 | 87.66 | 1464.8452 | 12 | 0.4 | 733.4302 | 2 | 89.35 |  |
| R.EVVEEEKKK.K | Y | 99.9 | 86.78 | 1116.6025 | 9 | -2.8 | 559.3070 | 2 | 10.08 |  |
| K.DAPKEEPPKEAA.A | Y | 99.9 | 86.66 | 1280.6248 | 12 | -1.5 | 641.3187 | 2 | 34.49 |  |
| R.AGDAKPDGAGELDFSGLLKK.R | Y | 99.9 | 86.48 | 1988.0214 | 20 | 0.5 | 663.6814 | 3 | 102.82 |  |
| D.VEAPRQDSAGQ.S | Y | 99.9 | 85.35 | 1156.5472 | 11 | 0.4 | 579.2811 | 2 | 21.87 |  |
| L.SDSPGVSKNT.A | Y | 99.9 | 85.24 | 990.4618 | 10 | -3.7 | 496.2363 | 2 | 9.81 |  |
| L.VWDPPKYDGGQPVTGY.L | Y | 99.9 | 84.65 | 1777.8311 | 16 | -1.3 | 889.9216 | 2 | 37.68 |  |
| L.ESFKRAGDAKPDGAGELDFSGLLK.K | Y | 99.9 | 84.63 | 2507.2656 | 24 | -0.2 | 627.8235 | 4 | 103.84 |  |
| K.LVVEISDPDLPLKWF.K | Y | 99.9 | 83.33 | 1769.9603 | 15 | -0.2 | 885.9872 | 2 | 142.34 |  |
| E.YVPKQEPPKIH.L | Y | 99.9 | 83.08 | 1334.7346 | 11 | 0.3 | 668.3748 | 2 | 41.35 |  |
| L.VIDDVRPEDEGDY.T | Y | 99.9 | 82.59 | 1520.6631 | 13 | -0.7 | 761.3383 | 2 | 81.66 |  |
| K.VTNPAGEDVATVM(+15.99).L | Y | 99.9 | 82.39 | 1318.6074 | 13 | 0.3 | 660.3112 | 2 | 77.18 | Oxidation (M) |
| V.TIREIVEQPKIRLPRHL.R | Y | 99.9 | 82.03 | 2097.2534 | 17 | -1.0 | 525.3201 | 4 | 96.52 |  |
| L.ELHIGKVVLGDRGDYRLEVK.A | Y | 99.9 | 81.39 | 2295.2698 | 20 | -1.6 | 574.8238 | 4 | 100.01 |  |
| R.EVVEEEKKKK.K | Y | 99.9 | 81.21 | 1244.6975 | 10 | -2.9 | 623.3542 | 2 | 8.23 |  |
| C.VEGSDEWIPANKEPVER.C | Y | 99.9 | 81.08 | 1953.9431 | 17 | 0.6 | 652.3220 | 3 | 33.85 |  |
| M.LRVVDVPDPPEAVRVT.S | Y | 99.9 | 80.58 | 1760.9784 | 16 | 0.1 | 588.0001 | 3 | 98.89 |  |
| R.AGDAKPDGAGELDFSGLLKKREVVEEEK.K | Y | 99.9 | 80.38 | 2986.5247 | 28 | 1.0 | 747.6392 | 4 | 101.82 |  |
| F.NIDVEAPRQDSAGQSLESF.K | Y | 99.9 | 80.18 | 2061.9602 | 19 | 0.3 | 1031.9877 | 2 | 33.98 |  |
| K.LVVEISDPDLPLK.W | Y | 99.9 | 78.96 | 1436.8126 | 13 | 0.2 | 719.4137 | 2 | 111.85 |  |
| K.GKDAPKEEPPK.E | Y | 99.9 | 78.36 | 1194.6244 | 11 | -1.0 | 598.3188 | 2 | 11.31 |  |
| L.VVEISDPDLPLKWF.K | Y | 99.9 | 78.30 | 1656.8762 | 14 | 2.0 | 829.4470 | 2 | 134.19 |  |
| A.AAEPPKEAPPEDQSPTAEEPT.G | Y | 99.9 | 77.70 | 2189.9963 | 21 | -1.4 | 1096.0039 | 2 | 36.60 |  |
| D.APKEEPPKEA.A | Y | 99.9 | 77.68 | 1094.5607 | 10 | -1.1 | 548.2870 | 2 | 10.19 |  |
| E.ISDPDLPLKW.F | Y | 99.9 | 77.46 | 1182.6284 | 10 | -0.4 | 592.3212 | 2 | 113.08 |  |
| R.VVDVPDPPEAV.R | Y | 99.9 | 76.74 | 1135.5760 | 11 | 0.2 | 568.7954 | 2 | 96.17 |  |
| L.NGKELPAKPT.I | Y | 99.9 | 76.65 | 1053.5818 | 10 | -0.1 | 527.7981 | 2 | 34.45 |  |
| C.SFNIDVEAPRQDSAGQ.S | Y | 99.9 | 76.59 | 1732.8016 | 16 | 1.5 | 867.4094 | 2 | 92.19 |  |
| L.KTGITFKPF.E | Y | 99.9 | 76.35 | 1037.5909 | 9 | -0.3 | 519.8026 | 2 | 33.98 |  |
| A.GDAKPDGAGELDFSGL.L | Y | 99.9 | 76.27 | 1547.7103 | 16 | -2.3 | 774.8607 | 2 | 105.91 |  |
| L.FVKEPPVLIVTPL.E | Y | 99.9 | 76.25 | 1450.8799 | 13 | 0.1 | 726.4473 | 2 | 120.93 |  |
| Q.IENM(+15.99)KDTATVR.I | Y | 99.9 | 75.79 | 1292.6394 | 11 | 0.1 | 647.3270 | 2 | 30.71 | Oxidation (M) |
| K.LNGKELPAKPT.I | Y | 99.9 | 75.50 | 1166.6659 | 11 | -0.8 | 584.3398 | 2 | 34.58 |  |
| V.EISDPDLPLKWF.K | Y | 99.9 | 75.24 | 1458.7394 | 12 | 0.8 | 730.3776 | 2 | 131.15 |  |
| A.ERGDEGRYT.I | Y | 99.9 | 74.76 | 1081.4788 | 9 | -1.0 | 541.7461 | 2 | 18.31 |  |
| E.KKKKKDDDDLGIPPEIW.E | Y | 99.9 | 74.76 | 2024.0941 | 17 | -1.2 | 1013.0531 | 2 | 38.06 |  |
| D.GAGELDFSGLLK.K | Y | 99.9 | 74.67 | 1205.6292 | 12 | -0.6 | 603.8215 | 2 | 34.14 |  |
| H.DFRSPPKFLTPL.I | Y | 99.9 | 74.08 | 1416.7765 | 12 | 1.4 | 709.3965 | 2 | 123.69 |  |
| E.LFVKEPPVLIVTPL.E | Y | 99.9 | 73.97 | 1563.9640 | 14 | 1.1 | 782.9901 | 2 | 129.92 |  |
| K.TGITFKPF.E | Y | 99.9 | 73.86 | 909.4960 | 8 | -0.3 | 455.7551 | 2 | 34.09 |  |
| T.YVRKVGEH.I | Y | 99.9 | 73.70 | 986.5297 | 8 | -0.5 | 494.2719 | 2 | 14.70 |  |
| S.MNTKPFMPIAPT.S | Y | 99.9 | 73.45 | 1346.6726 | 12 | 0.8 | 674.3441 | 2 | 100.11 |  |
| E.VSDEKVTGK.W | Y | 99.9 | 73.35 | 961.5080 | 9 | -1.9 | 481.7603 | 2 | 9.83 |  |
| S.M(+15.99)NTKPFMPIAPT.S | Y | 99.9 | 73.01 | 1362.6676 | 12 | 0.5 | 682.3414 | 2 | 93.87 | Oxidation (M) |
| F.KRAGDAKPDGAGEL.D | Y | 99.9 | 72.86 | 1383.7106 | 14 | -0.6 | 692.8621 | 2 | 34.51 |  |
| L.VVEISDPDLPLK.W | Y | 99.9 | 72.83 | 1323.7285 | 12 | 0.7 | 662.8720 | 2 | 101.94 |  |
| E.APPEDQSPTAEEPTGIFLKKPDSVSVETGKDAVIM(+15.99).A | Y | 99.9 | 72.80 | 3698.8235 | 35 | -2.5 | 925.7109 | 4 | 104.81 | Oxidation (M) |
| K.Q(-17.03)EPPKIH.L | Y | 99.9 | 72.34 | 830.4286 | 7 | -0.7 | 416.2213 | 2 | 34.66 | Pyro-glu from Q |
| E.SAERGDEGRYT.I | Y | 99.9 | 72.21 | 1239.5480 | 11 | 0.9 | 620.7818 | 2 | 23.81 |  |
| N.GKELPAKPT.I | Y | 99.9 | 71.69 | 939.5389 | 9 | 0.5 | 470.7769 | 2 | 32.93 |  |
| R.EVVEEEKKKKK.D | Y | 99.9 | 71.67 | 1372.7925 | 11 | -1.5 | 687.4025 | 2 | 7.15 |  |
| L.LKKREVVEEEKK.K | Y | 99.9 | 71.62 | 1513.8827 | 12 | -0.6 | 379.4777 | 4 | 33.21 |  |
| A.AEPPKEAPPEDQSPTAEEPTGIF.L | Y | 99.9 | 71.60 | 2436.1331 | 23 | -0.3 | 1219.0735 | 2 | 37.39 |  |
| L.KKPDSVSVETGKDAVIM(+15.99).A | Y | 99.9 | 71.50 | 1818.9397 | 17 | 1.0 | 607.3211 | 3 | 70.00 | Oxidation (M) |
| A.AAEPPKEAPPEDQ.S | Y | 99.9 | 71.40 | 1377.6411 | 13 | -1.9 | 689.8265 | 2 | 34.55 |  |
| F.NIDVEAPRQD.S | Y | 99.9 | 71.07 | 1155.5520 | 10 | 0.3 | 578.7834 | 2 | 69.83 |  |
| A.AAEPPKEAPPEDQSPTA.E | Y | 99.9 | 71.01 | 1733.8108 | 17 | 0.2 | 867.9128 | 2 | 65.83 |  |
| D.DVRPEDEGDYT.F | Y | 99.9 | 70.45 | 1294.5312 | 11 | -0.1 | 648.2728 | 2 | 66.58 |  |
| L.VIDDVRPE.D | Y | 99.9 | 70.36 | 941.4818 | 8 | -1.6 | 471.7474 | 2 | 34.69 |  |
| D.VEAPRQDSAGQSLESFKR.A | Y | 99.9 | 70.16 | 2004.0024 | 18 | -1.1 | 502.0073 | 4 | 33.75 |  |
| W.MKNKVEIH.E | Y | 99.9 | 70.07 | 997.5378 | 8 | -1.0 | 499.7757 | 2 | 26.07 |  |
| L.ERKKKGSQRW.M | Y | 99.9 | 69.97 | 1301.7316 | 10 | -1.4 | 434.9172 | 3 | 34.03 |  |
| K.DAPKEEPPKEA.A | Y | 99.9 | 69.81 | 1209.5876 | 11 | -0.7 | 605.8007 | 2 | 34.45 |  |
| E.ISDPDLPLK.W | Y | 99.9 | 69.54 | 996.5491 | 9 | 0.2 | 499.2819 | 2 | 87.65 |  |
| K.NTAHILK.T | Y | 99.9 | 69.53 | 795.4603 | 7 | -0.7 | 398.7371 | 2 | 25.38 |  |
| R.AGDAKPDGAGELDFSGLLKKREVVEEEKK.K | Y | 99.9 | 68.94 | 3114.6196 | 29 | -1.5 | 623.9302 | 5 | 98.37 |  |
| L.DFSGLLKKR.E | Y | 99.9 | 68.54 | 1062.6185 | 9 | 2.4 | 532.3178 | 2 | 99.92 |  |
| R.AGDAKPDGAGELDFSGLL.K | Y | 99.9 | 68.54 | 1731.8314 | 18 | 0.7 | 866.9236 | 2 | 118.57 |  |
| H.DFRSPPKFL.T | Y | 99.9 | 68.49 | 1105.5919 | 9 | 0.2 | 553.8033 | 2 | 97.27 |  |
| L.KKPDSVSVETGKDAVIM.A | Y | 99.9 | 68.32 | 1802.9447 | 17 | -0.2 | 601.9887 | 3 | 79.55 |  |
| Q.VDRGNKVKLVVEISDPDLPLKWF.K | Y | 99.9 | 68.27 | 2666.4795 | 23 | 4.6 | 889.8379 | 3 | 127.40 |  |
| E.VNLEDRGHYQ.V | Y | 99.9 | 67.56 | 1229.5789 | 10 | -0.7 | 615.7963 | 2 | 62.66 |  |
| Q.SLESFKRAGDAKPDGAGELDFSGLLKKR.E | Y | 99.9 | 67.50 | 2991.5776 | 28 | -3.2 | 748.8993 | 4 | 102.27 |  |
| V.SQPSM(+15.99)NTKPFMPIAPT.S | Y | 99.9 | 67.30 | 1761.8430 | 16 | 0.2 | 881.9290 | 2 | 95.88 | Oxidation (M) |
| K.KREVVEEEKK.K | Y | 99.9 | 67.13 | 1272.7037 | 10 | -1.6 | 637.3582 | 2 | 11.77 |  |
| Y.LVQKADKKTM.E | Y | 99.9 | 67.11 | 1160.6587 | 10 | 0.3 | 581.3368 | 2 | 15.25 |  |
| R.EVVEEEKK.K | Y | 99.9 | 67.07 | 988.5076 | 8 | -2.6 | 495.2598 | 2 | 10.72 |  |
| A.AEPPKEAPPEDQSPT.A | Y | 99.9 | 67.05 | 1591.7366 | 15 | 0.5 | 796.8760 | 2 | 63.01 |  |
| V.VEISDPDLPLK.W | Y | 99.9 | 66.84 | 1224.6602 | 11 | 0.1 | 613.3374 | 2 | 96.86 |  |
| S.DFDTVFFVR.E | Y | 99.9 | 66.61 | 1144.5553 | 9 | 1.9 | 573.2860 | 2 | 118.39 |  |
| R.AGDAKPDGAGEL.D | Y | 99.9 | 66.58 | 1099.5145 | 12 | -0.9 | 550.7640 | 2 | 33.76 |  |
| K.KREVVEEEK.K | Y | 99.9 | 66.43 | 1144.6088 | 9 | -0.7 | 573.3113 | 2 | 13.18 |  |
| K.VTNPAGEDVAT.V | Y | 99.9 | 66.26 | 1072.5037 | 11 | -1.0 | 537.2585 | 2 | 34.72 |  |
| R.LDVRVPQ | Y | 99.9 | 66.11 | 825.4708 | 7 | 0.1 | 413.7427 | 2 | 33.85 |  |
| K.KPDSVSVETGK.D | Y | 99.9 | 65.87 | 1145.5928 | 11 | -0.1 | 382.8715 | 3 | 31.85 |  |
| Y.KEHDFRSPPKFLTPL.I | Y | 99.9 | 65.75 | 1810.9729 | 15 | -1.6 | 604.6639 | 3 | 96.48 |  |
| L.GSKSGARF.S | Y | 99.9 | 65.44 | 808.4191 | 8 | -0.4 | 405.2167 | 2 | 13.60 |  |
| K.EHDFRSPPKFLTPL.I | Y | 99.9 | 65.30 | 1682.8779 | 14 | 1.2 | 561.9673 | 3 | 102.67 |  |
| S.MNTKPFM(+15.99)PIAPT.S | Y | 99.9 | 65.20 | 1362.6676 | 12 | 0.5 | 682.3414 | 2 | 90.38 | Oxidation (M) |
| L.VWDPPKYDGGQPVT.G | Y | 99.9 | 65.16 | 1557.7463 | 14 | 0.1 | 779.8805 | 2 | 90.78 |  |
| A.AAEPPKEAPPEDQSPTAEEPTGIF.L | Y | 99.9 | 65.11 | 2507.1702 | 24 | -0.5 | 836.7302 | 3 | 100.99 |  |
| Q.IENM(+15.99)KDTAT.V | Y | 99.9 | 65.01 | 1037.4700 | 9 | -1.4 | 519.7415 | 2 | 6.52 | Oxidation (M) |
| L.LKKREVVEEE.K | Y | 99.9 | 64.99 | 1257.6929 | 10 | -0.7 | 629.8533 | 2 | 30.85 |  |
| V.EISDPDLPLK.W | Y | 99.9 | 64.87 | 1125.5917 | 10 | -1.1 | 563.8025 | 2 | 88.63 |  |
| L.EDQQVFVGDRVEMS.V | Y | 99.9 | 64.55 | 1637.7355 | 14 | 0.4 | 819.8754 | 2 | 93.35 |  |
| N.LEDRGHYQ.V | Y | 99.9 | 64.35 | 1016.4675 | 8 | -0.4 | 509.2408 | 2 | 22.49 |  |
| T.GEPPPVATW.M | Y | 99.9 | 63.86 | 952.4654 | 9 | -0.3 | 477.2398 | 2 | 111.86 |  |
| V.DVPDPPEAV.R | Y | 99.9 | 63.82 | 937.4392 | 9 | -1.1 | 469.7264 | 2 | 82.13 |  |
| K.VTNPAGEDVATVM.L | Y | 99.9 | 63.77 | 1302.6125 | 13 | -0.5 | 652.3132 | 2 | 34.01 |  |
| A.GDAKPDGAGEL.D | Y | 99.9 | 63.57 | 1028.4774 | 11 | -1.2 | 515.2454 | 2 | 34.70 |  |
| A.AAEPPKEAPPEDQSPT.A | Y | 99.9 | 63.55 | 1662.7737 | 16 | 0.1 | 832.3942 | 2 | 62.88 |  |
| E.IHEDPKFL.M | Y | 99.9 | 63.21 | 997.5233 | 8 | -1.5 | 499.7682 | 2 | 77.26 |  |
| K.LVVEISDPDLPLKWFKN.G | Y | 99.9 | 63.00 | 2012.0981 | 17 | -0.5 | 671.7063 | 3 | 126.51 |  |
| K.VTNPAGEDV.A | Y | 99.9 | 62.90 | 900.4188 | 9 | 0.2 | 451.2168 | 2 | 67.91 |  |
| K.VTNPAGEDVA.T | Y | 99.9 | 62.79 | 971.4559 | 10 | -0.1 | 486.7352 | 2 | 64.59 |  |
| L.VEWQPPKDNGNSEIT.G | Y | 99.9 | 62.69 | 1712.8005 | 15 | -0.2 | 857.4073 | 2 | 86.14 |  |
| F.KNGQEIKPS.L | Y | 99.9 | 62.68 | 999.5349 | 9 | -0.4 | 500.7745 | 2 | 10.52 |  |
| Y.LLERKKKGSQRW.M | Y | 99.9 | 62.60 | 1527.8998 | 12 | -0.5 | 510.3069 | 3 | 34.43 |  |
| E.TGKDAVIM(+15.99).A | Y | 99.9 | 62.58 | 849.4266 | 8 | -1.0 | 425.7201 | 2 | 30.81 | Oxidation (M) |
| F.NIDVEAPRQDSAGQSLESFKR.A | Y | 99.9 | 62.46 | 2346.1562 | 21 | 1.1 | 587.5470 | 4 | 88.73 |  |
| K.SEYERIAF.Q | Y | 99.9 | 62.41 | 1013.4818 | 8 | -1.3 | 507.7475 | 2 | 92.54 |  |
| E.EKKKKKDDDDLGIPPEIW.E | Y | 99.9 | 62.37 | 2153.1367 | 18 | 0.5 | 539.2917 | 4 | 101.16 |  |
| R.EIVEQPKIRLPRHL.R | Y | 99.9 | 62.26 | 1727.0205 | 14 | -0.2 | 864.5173 | 2 | 38.13 |  |
| N.TKPFMPIAPT.S | Y | 99.9 | 62.24 | 1101.5892 | 10 | 0.0 | 551.8019 | 2 | 95.59 |  |
| S.HVGRF.H | Y | 99.9 | 62.19 | 614.3289 | 5 | -1.1 | 308.1714 | 2 | 27.20 |  |
| V.WDPPKYDGGQPVTGY.L | Y | 99.9 | 61.97 | 1678.7627 | 15 | 0.8 | 840.3893 | 2 | 97.92 |  |
| N.TKPFM(+15.99)PIAPT.S | Y | 99.9 | 61.87 | 1117.5841 | 10 | -0.7 | 559.7990 | 2 | 79.74 | Oxidation (M) |
| L.NIRRPSPF.D | Y | 99.9 | 61.76 | 985.5457 | 8 | -0.3 | 493.7800 | 2 | 69.60 |  |
| K.YVFENVGKK.R | Y | 99.9 | 61.54 | 1082.5760 | 9 | -0.7 | 542.2949 | 2 | 70.44 |  |
| R.IEQRPDSS.S | Y | 99.9 | 60.96 | 930.4407 | 8 | -0.9 | 466.2272 | 2 | 7.32 |  |
| F.TKKLDPAYQ.V | Y | 99.9 | 60.83 | 1062.5709 | 9 | -0.8 | 532.2923 | 2 | 34.53 |  |
| V.IDDVRPEDEGDYT.F | Y | 99.9 | 60.59 | 1522.6423 | 13 | 0.0 | 762.3284 | 2 | 75.35 |  |
| L.GDRGDYRL.E | Y | 99.9 | 60.46 | 950.4570 | 8 | -0.4 | 476.2356 | 2 | 34.66 |  |
| Y.SEVNLEDR.G | Y | 99.9 | 60.04 | 960.4512 | 8 | -0.3 | 481.2328 | 2 | 62.22 |  |
| E.ISDPDLPLKWFKNGQEIKPS.L | Y | 99.9 | 59.98 | 2311.2212 | 20 | 0.3 | 771.4146 | 3 | 110.27 |  |
| I.LVWDPPKYDGGQPVTGY.L | Y | 99.9 | 59.88 | 1890.9152 | 17 | 0.1 | 946.4649 | 2 | 108.28 |  |
| L.LERKKKGSQRW.M | Y | 99.9 | 59.13 | 1414.8157 | 11 | -2.4 | 354.7104 | 4 | 34.17 |  |
| S.M(+15.99)NTKPFM(+15.99)PIAPT.S | Y | 99.9 | 59.02 | 1378.6625 | 12 | -0.5 | 690.3381 | 2 | 81.40 | Oxidation (M) |
| R.EIVEQPKIRLPR.H | Y | 99.9 | 58.96 | 1476.8776 | 12 | -0.3 | 739.4458 | 2 | 39.34 |  |
| D.VEAPRQD.S | Y | 99.9 | 58.51 | 813.3981 | 7 | -0.9 | 407.7059 | 2 | 11.39 |  |
| E.YVPKQEPPKIHL.D | Y | 99.9 | 58.47 | 1447.8187 | 12 | -1.4 | 483.6129 | 3 | 80.67 |  |
| Y.LLERKKKGSQ.R | Y | 99.9 | 58.09 | 1185.7194 | 10 | -0.7 | 593.8665 | 2 | 19.63 |  |
| A.YQVDRGNKVK.L | Y | 99.9 | 57.98 | 1205.6516 | 10 | -0.3 | 603.8329 | 2 | 28.55 |  |
| S.ITGEPPPVATW.M | Y | 99.9 | 57.83 | 1166.5972 | 11 | -0.9 | 584.3054 | 2 | 108.06 |  |
| L.LKKREVVEE.E | Y | 99.9 | 57.80 | 1128.6503 | 9 | -0.6 | 565.3321 | 2 | 24.76 |  |
| K.KREVVEEEKKK.K | Y | 99.9 | 57.76 | 1400.7987 | 11 | -0.9 | 701.4060 | 2 | 12.18 |  |
| R.KAPKGKDAPK.E | Y | 99.9 | 57.44 | 1038.6185 | 10 | -4.4 | 520.3143 | 2 | 5.95 |  |
| L.FVKEPPVLIV.T | Y | 99.9 | 57.44 | 1139.6954 | 10 | 0.2 | 570.8551 | 2 | 108.54 |  |
| N.EYYFR.V | N | 99.9 | 57.36 | 776.3493 | 5 | -0.3 | 389.1818 | 2 | 73.64 |  |
| S.EYERIAF.Q | Y | 99.9 | 57.20 | 926.4497 | 7 | -0.2 | 464.2321 | 2 | 36.64 |  |
| S.HVGRFH.K | Y | 99.9 | 57.13 | 751.3878 | 6 | 0.0 | 376.7012 | 2 | 19.09 |  |
| L.KWRPPDRIGAG.G | Y | 99.9 | 56.93 | 1251.6836 | 11 | -1.2 | 626.8483 | 2 | 37.46 |  |
| G.KVVLGDRGDYRL.E | Y | 99.9 | 56.88 | 1389.7728 | 12 | -2.1 | 348.4497 | 4 | 80.55 |  |
| Q.VDRGNKVKLVVEISDPDLPLKWFKNGQEIKPS.L | Y | 99.9 | 56.83 | 3648.0039 | 32 | 0.1 | 730.6082 | 5 | 116.28 |  |
| A.YQVDRGN.K | Y | 99.9 | 56.66 | 850.3933 | 7 | -0.9 | 426.2036 | 2 | 20.69 |  |
| L.VWDPPKYD.G | Y | 99.9 | 56.59 | 1018.4760 | 8 | 0.3 | 510.2454 | 2 | 82.54 |  |
| V.SQPSM(+15.99)NTKPFM(+15.99)PIAPT.S | Y | 99.9 | 56.47 | 1777.8379 | 16 | 0.3 | 889.9265 | 2 | 83.05 | Oxidation (M) |
| V.WTKGGAPVDTS.R | Y | 99.9 | 56.36 | 1117.5404 | 11 | -0.9 | 559.7770 | 2 | 61.68 |  |
| K.KREVVEEEKKKKKDDDDLGIPPEIW.E | Y | 99.9 | 56.34 | 3022.5974 | 25 | -1.0 | 756.6559 | 4 | 93.76 |  |
| D.PPKYDGGQPVTGY.L | Y | 99.9 | 56.00 | 1377.6565 | 13 | -0.1 | 689.8354 | 2 | 77.02 |  |
| Y.LVQKADKK.T | Y | 99.9 | 55.99 | 928.5706 | 8 | -3.1 | 465.2911 | 2 | 6.78 |  |
| A.LVEWQPPKDN.G | Y | 99.9 | 55.93 | 1224.6139 | 10 | 0.1 | 613.3143 | 2 | 86.26 |  |
| E.LFVKEPPVLIV.T | Y | 99.9 | 55.87 | 1252.7794 | 11 | -0.7 | 627.3965 | 2 | 119.55 |  |
| Y.LLERKKKGSQR.W | Y | 99.9 | 55.71 | 1341.8204 | 11 | -0.4 | 448.2806 | 3 | 20.88 |  |
| K.GKDAPKEEPPKEAAAEPPKEAPPEDQSPTAEEPTGIFLKKPDSVSVETGKDAVIM(+15.99).A | Y | 99.9 | 55.34 | 5797.8770 | 55 | 1.2 | 967.3213 | 6 | 97.89 | Oxidation (M) |
| E.TGKDAVIM.A | Y | 99.9 | 55.23 | 833.4317 | 8 | -1.1 | 417.7227 | 2 | 34.79 |  |
| Y.YFRVY.T | Y | 99.9 | 55.17 | 746.3751 | 5 | -0.6 | 374.1946 | 2 | 85.77 |  |
| R.KAPKGKDAPKEEPPKEAAAEPPKEAPPEDQSPTAEEPTGIFLKKPDSVSVETGKDAVIM(+15.99)AK.L | Y | 99.9 | 55.04 | 6421.2886 | 61 | -0.9 | 714.4832 | 9 | 94.14 | Oxidation (M) |
| F.KRAGDAKPDGAGELDFSGL.L | Y | 99.9 | 54.77 | 1902.9435 | 19 | 0.6 | 635.3221 | 3 | 94.67 |  |
| V.VNDEKC(+57.02)FTEL.F | Y | 99.9 | 54.24 | 1253.5598 | 10 | 0.5 | 627.7875 | 2 | 87.43 | Carbamidomethylation |
| A.LVEWQPPKDNGNSEIT.G | Y | 99.9 | 54.20 | 1825.8846 | 16 | 0.0 | 913.9496 | 2 | 94.80 |  |
| A.GRSEPATLVQPVTIR.E | Y | 99.9 | 54.08 | 1622.9104 | 15 | 1.9 | 541.9785 | 3 | 87.02 |  |
| R.HLRQTY.V | Y | 99.9 | 53.93 | 816.4242 | 6 | -1.3 | 409.2188 | 2 | 22.15 |  |
| A.KKSEYERIAF.Q | Y | 99.9 | 53.90 | 1269.6716 | 10 | -0.7 | 424.2309 | 3 | 74.64 |  |
| K.DDDDLGIPPEIW.E | Y | 99.9 | 53.87 | 1383.6194 | 12 | -0.1 | 692.8169 | 2 | 175.83 |  |
| K.EHDFRSPPKFL.T | Y | 99.9 | 53.76 | 1371.6935 | 11 | 0.1 | 458.2385 | 3 | 88.25 |  |
| R.GHPKPKVV.W | Y | 99.9 | 53.62 | 860.5232 | 8 | -0.4 | 431.2687 | 2 | 19.19 |  |
| L.VIDDVRPEDEGDYTF.V | Y | 99.9 | 53.50 | 1768.7791 | 15 | 1.8 | 885.3984 | 2 | 100.17 |  |
| Y.VRKVGEHIN.L | Y | 99.9 | 53.43 | 1050.5934 | 9 | -1.2 | 526.3033 | 2 | 21.50 |  |
| Y.VFENVGKKR.I | Y | 99.9 | 53.17 | 1075.6138 | 9 | -0.4 | 359.5450 | 3 | 34.45 |  |
| A.GNKLRLDVSIT.G | Y | 99.9 | 53.06 | 1214.6982 | 11 | -0.1 | 608.3563 | 2 | 91.00 |  |
| E.WQPPKDNGNSEIT.G | Y | 99.9 | 53.01 | 1484.6896 | 13 | -1.0 | 743.3513 | 2 | 38.83 |  |
| W.TKGGAPVDTS.R | Y | 99.9 | 52.96 | 931.4611 | 10 | -0.3 | 466.7377 | 2 | 11.85 |  |
| K.EAPPEDQSPTAEEPTGIF.L | Y | 99.9 | 52.83 | 1913.8530 | 18 | 0.2 | 957.9340 | 2 | 104.51 |  |
| A.GNKLRLD.V | Y | 99.9 | 52.83 | 814.4661 | 7 | -1.4 | 408.2397 | 2 | 34.61 |  |
| F.Q(-17.03)GKPRPQ.V | Y | 99.9 | 52.60 | 792.4242 | 7 | -3.8 | 397.2179 | 2 | 5.12 | Pyro-glu from Q |
| L.KKPDSVSVE.T | Y | 99.9 | 52.44 | 987.5236 | 9 | -0.1 | 494.7690 | 2 | 25.69 |  |
| L.VEWQPPKDN.G | Y | 99.9 | 52.28 | 1111.5298 | 9 | -1.5 | 556.7714 | 2 | 72.43 |  |
| Y.RFKKDGKRHIL.I | Y | 99.9 | 52.05 | 1396.8414 | 11 | -0.5 | 350.2175 | 4 | 34.37 |  |
| F.TKKLDPA.Y | Y | 99.9 | 52.04 | 771.4490 | 7 | -0.6 | 386.7316 | 2 | 9.84 |  |
| N.GKELPAKPTIK.W | Y | 99.9 | 52.02 | 1180.7179 | 11 | -0.6 | 394.5797 | 3 | 34.54 |  |
| L.FVKEPPVLIVTPLEDQQVFVGDRVEMS.V | Y | 99.9 | 51.98 | 3070.6047 | 27 | -0.2 | 1024.5420 | 3 | 126.75 |  |
| R.KVGEHIN.L | Y | 99.9 | 51.93 | 795.4239 | 7 | -0.3 | 398.7191 | 2 | 12.05 |  |
| R.KVGEHINL.V | Y | 99.9 | 51.85 | 908.5079 | 8 | -0.2 | 455.2612 | 2 | 68.36 |  |
| T.GKWYKN.G | Y | 99.9 | 51.63 | 794.4075 | 6 | -1.6 | 398.2104 | 2 | 33.52 |  |
| L.GDRGDYR.L | Y | 99.9 | 51.57 | 837.3729 | 7 | -0.6 | 419.6935 | 2 | 7.81 |  |
| K.ELPAKPTIK.W | Y | 99.9 | 51.57 | 995.6015 | 9 | -0.3 | 498.8079 | 2 | 63.54 |  |
| R.VVDVPDPPEAVRVT.S | Y | 99.9 | 51.56 | 1491.7932 | 14 | 1.9 | 746.9053 | 2 | 95.34 |  |
| L.LKKREVVEEEKKK.K | Y | 99.9 | 51.53 | 1641.9777 | 13 | -1.4 | 411.5011 | 4 | 33.07 |  |
| K.GKDAPKEEPPKEAAAEPPKEAPPEDQSPTAEEPTGIF.L | Y | 99.9 | 51.44 | 3883.8638 | 37 | 0.3 | 971.9735 | 4 | 91.20 |  |
| M.NTKPFM(+15.99)PIAPT.S | Y | 99.9 | 51.32 | 1231.6271 | 11 | 0.3 | 616.8210 | 2 | 82.04 | Oxidation (M) |
| Q.VDRGNKVKL.V | Y | 99.9 | 51.24 | 1027.6138 | 9 | -0.5 | 343.5450 | 3 | 34.48 |  |
| T.KKLDPAYQ.V | Y | 99.9 | 50.89 | 961.5233 | 8 | -1.1 | 481.7684 | 2 | 34.51 |  |
| A.AAEPPKEAPPEDQSPTAEEPTGIFL.K | Y | 99.9 | 50.66 | 2620.2544 | 25 | 0.6 | 874.4260 | 3 | 109.30 |  |
| T.IKVTNPAGEDV.A | Y | 99.9 | 50.60 | 1141.5979 | 11 | 0.5 | 571.8065 | 2 | 75.85 |  |
| M.WM(+15.99)KDGVELT.R | Y | 99.9 | 50.47 | 1093.5114 | 9 | -0.2 | 547.7628 | 2 | 83.24 | Oxidation (M) |
| N.IDVEAPRQD.S | Y | 99.9 | 50.43 | 1041.5090 | 9 | -0.5 | 521.7615 | 2 | 65.77 |  |
| Q.SLESFKR.A | Y | 99.9 | 50.24 | 865.4657 | 7 | -2.1 | 433.7392 | 2 | 35.93 |  |
| L.KKREVVEEEKKKKKDDDDLGIPPEIW.E | Y | 99.9 | 50.22 | 3150.6924 | 26 | 0.2 | 788.6805 | 4 | 90.54 |  |
| L.FVKEPPVL.I | Y | 99.9 | 50.07 | 927.5429 | 8 | 0.0 | 464.7787 | 2 | 89.33 |  |
| M.LRVVDVPDPPE.A | Y | 99.9 | 50.02 | 1234.6558 | 11 | 0.7 | 618.3356 | 2 | 91.17 |  |
| L.VWDPPKYDGGQPV.T | Y | 99.9 | 50.00 | 1456.6986 | 13 | -1.6 | 729.3554 | 2 | 92.37 |  |
| A.ARSDSGEYELS.V | Y | 99.9 | 49.75 | 1212.5259 | 11 | 0.4 | 607.2704 | 2 | 34.73 |  |
| K.QEPPKIH.L | Y | 99.9 | 49.50 | 847.4552 | 7 | -0.5 | 424.7347 | 2 | 33.28 |  |
| Y.LLERKKKGS.Q | Y | 99.9 | 49.48 | 1057.6608 | 9 | 0.0 | 529.8376 | 2 | 13.10 |  |
| S.EVNLEDRGHYQ.V | Y | 99.9 | 49.46 | 1358.6215 | 11 | 0.7 | 680.3185 | 2 | 66.90 |  |
| K.NKVEIHEDPK.F | Y | 99.8 | 49.39 | 1207.6196 | 10 | -0.5 | 604.8168 | 2 | 25.41 |  |
| N.GVEVRPS.K | Y | 99.9 | 49.30 | 742.3973 | 7 | -0.6 | 372.2057 | 2 | 29.19 |  |
| K.VTNPAGEDVATV.M | Y | 99.9 | 49.03 | 1171.5720 | 12 | 0.3 | 586.7935 | 2 | 82.88 |  |
| A.NKEPVERCG.F | Y | 99.9 | 49.01 | 1030.4866 | 9 | 0.0 | 516.2505 | 2 | 7.92 |  |
| R.KAPKGKDAPKEEPPKEAAAEPPKEAPPEDQSPTAEEPTGIFLKKPDSVSVETGK.D | Y | 99.8 | 48.95 | 5676.9048 | 54 | 2.6 | 710.6223 | 8 | 88.81 |  |
| F.VGDRVEM(+15.99)S.V | Y | 99.9 | 48.66 | 907.4069 | 8 | -1.2 | 454.7102 | 2 | 8.04 | Oxidation (M) |
| V.EWQPPKDNGNSEIT.G | Y | 99.9 | 48.65 | 1613.7322 | 14 | -1.3 | 807.8723 | 2 | 81.32 |  |
| K.LVVEISDPDLPLKW.F | Y | 99.9 | 48.57 | 1622.8918 | 14 | 0.4 | 812.4536 | 2 | 128.34 |  |
| E.IVEQPKIRLPRHL.R | Y | 99.9 | 48.57 | 1597.9780 | 13 | -0.9 | 400.5014 | 4 | 87.33 |  |
| F.LKKPDSVSVE.T | Y | 99.9 | 48.45 | 1100.6077 | 10 | -1.0 | 551.3105 | 2 | 59.89 |  |
| M.WMKDGVELT.R | Y | 99.9 | 48.35 | 1077.5165 | 9 | 0.0 | 539.7655 | 2 | 88.34 |  |
| V.FENVGKKR.I | Y | 99.9 | 48.34 | 976.5453 | 8 | -1.0 | 489.2795 | 2 | 34.45 |  |
| K.KLDPAYQ.V | Y | 99.9 | 48.33 | 833.4283 | 7 | -0.2 | 417.7213 | 2 | 56.66 |  |
| K.KSAAFTK.K | Y | 99.8 | 48.30 | 751.4228 | 7 | -1.0 | 376.7183 | 2 | 12.77 |  |
| N.AIGVSQPSM(+15.99)NTKPFM(+15.99)PIAPTSAPQHL.T | Y | 99.8 | 48.27 | 2751.3723 | 26 | 1.2 | 918.1325 | 3 | 100.02 | Oxidation (M) |
| F.LKKPDSVS.V | Y | 99.9 | 48.25 | 872.4967 | 8 | -1.1 | 437.2552 | 2 | 24.79 |  |
| R.EVVEEEKKKKKD.D | Y | 99.9 | 48.21 | 1487.8195 | 12 | -1.8 | 744.9156 | 2 | 6.93 |  |
| S.Q(-17.03)PSM(+15.99)NTKPFM(+15.99)PIAPT.S | Y | 99.9 | 48.10 | 1673.7793 | 15 | -0.4 | 837.8966 | 2 | 95.04 | Pyro-glu from Q; Oxidation (M) |
| S.Q(-17.03)PSM(+15.99)NTKPFMPIAPT.S | Y | 99.9 | 48.08 | 1657.7844 | 15 | -0.5 | 829.8991 | 2 | 105.48 | Pyro-glu from Q; Oxidation (M) |
| N.GQEIKPS.L | Y | 99.9 | 48.03 | 757.3970 | 7 | -1.6 | 379.7052 | 2 | 43.59 |  |
| A.TLVQPVTIR.E | Y | 99.9 | 47.94 | 1025.6233 | 9 | -0.1 | 513.8188 | 2 | 86.49 |  |
| F.VKEPPVLIVTPL.E | Y | 99.9 | 47.87 | 1303.8115 | 12 | -0.1 | 652.9130 | 2 | 116.53 |  |
| R.FKKDGKRHIL.I | Y | 99.9 | 47.85 | 1240.7404 | 10 | -0.4 | 311.1922 | 4 | 34.37 |  |
| V.LGDRGDYRL.E | Y | 99.9 | 47.75 | 1063.5410 | 9 | -0.4 | 532.7776 | 2 | 72.51 |  |
| S.AERGDEGRYT.I | Y | 99.9 | 47.69 | 1152.5159 | 10 | -0.2 | 577.2651 | 2 | 21.39 |  |
| A.PKEEPPKEA.A | Y | 99.9 | 47.63 | 1023.5236 | 9 | -1.2 | 512.7685 | 2 | 25.77 |  |
| N.YRFKKD.G | Y | 99.9 | 47.60 | 855.4603 | 6 | -0.4 | 428.7372 | 2 | 25.10 |  |
| M.L(+27.99)RVVDVPDPPEAV.R | Y | 99.9 | 47.56 | 1432.7561 | 13 | 3.4 | 717.3878 | 2 | 115.70 | Formylation |
| L.KWRPPDRIGAGGIDGY.L | Y | 99.9 | 47.47 | 1756.9009 | 16 | 0.2 | 586.6410 | 3 | 93.03 |  |
| K.SNYRFK.K | Y | 99.9 | 47.41 | 813.4133 | 6 | -1.3 | 407.7134 | 2 | 34.54 |  |
| K.GKDAPKEEPPKEAAAEPPKEAPPEDQSPTAEEPTGIFLKKPDSVSVETGKDAVIM(+15.99)AK.L | Y | 99.8 | 47.41 | 5997.0088 | 57 | 4.5 | 857.7267 | 7 | 96.34 | Oxidation (M) |
| F.LKKPDSVSVETGK.D | Y | 99.8 | 47.40 | 1386.7719 | 13 | -0.3 | 463.2644 | 3 | 33.58 |  |
| L.LKKREVVEEEKKKKKDDDDLGIPPEIW.E | Y | 99.9 | 47.31 | 3263.7764 | 27 | -3.0 | 653.7606 | 5 | 92.75 |  |
| L.FRVIGVN.I | Y | 99.9 | 47.21 | 803.4653 | 7 | 0.1 | 402.7400 | 2 | 36.01 |  |
| L.IDRVVVA.G | Y | 99.9 | 47.01 | 770.4650 | 7 | 0.2 | 386.2399 | 2 | 39.06 |  |
| K.LNGKELPAKPTIK.W | Y | 99.8 | 46.94 | 1407.8448 | 13 | -1.4 | 470.2882 | 3 | 67.19 |  |
| T.LNIRRPSPF.D | Y | 99.9 | 46.91 | 1098.6298 | 9 | -1.5 | 367.2166 | 3 | 85.99 |  |
| R.EIVEQPK.I | Y | 99.8 | 46.90 | 841.4545 | 7 | 0.3 | 421.7346 | 2 | 33.61 |  |
| S.FKETHDAA.S | Y | 99.9 | 46.59 | 917.4243 | 8 | -0.4 | 459.7192 | 2 | 8.69 |  |
| L.VQPVTIR.E | Y | 99.9 | 46.22 | 811.4916 | 7 | -1.7 | 406.7524 | 2 | 36.91 |  |
| K.KKDDDDLGIPPEIW.E | Y | 99.9 | 46.20 | 1639.8093 | 14 | 1.5 | 820.9132 | 2 | 112.70 |  |
| F.NIDVEAPRQDSA.G | Y | 99.9 | 46.10 | 1313.6211 | 12 | 1.0 | 657.8185 | 2 | 72.32 |  |
| H.EDPKFLM.T | Y | 99.9 | 46.01 | 878.4208 | 7 | -0.2 | 440.2176 | 2 | 93.98 |  |
| K.GKDAPKEEPPKEAA.A | Y | 99.7 | 45.79 | 1465.7412 | 14 | -1.3 | 733.8770 | 2 | 31.22 |  |
| S.EYERIAFQ.Y | Y | 99.9 | 45.78 | 1054.5083 | 8 | 0.0 | 528.2614 | 2 | 83.47 |  |
| N.IAGRSEPATLVQPVTIR.E | Y | 99.7 | 45.49 | 1807.0315 | 17 | -2.3 | 603.3497 | 3 | 90.69 |  |
| L.VWDPPKYDGGQPVTGYL.L | Y | 99.9 | 45.46 | 1890.9152 | 17 | 0.0 | 946.4648 | 2 | 110.66 |  |
| L.ESFKR.A | Y | 99.7 | 45.27 | 665.3496 | 5 | -0.2 | 333.6820 | 2 | 33.38 |  |
| G.SKSGARF.S | Y | 99.9 | 45.21 | 751.3976 | 7 | -1.4 | 376.7056 | 2 | 15.05 |  |
| F.QGKPRPQ.V | Y | 99.9 | 45.05 | 809.4507 | 7 | -4.2 | 405.7310 | 2 | 5.09 |  |
| F.KKDGKRHIL.I | Y | 99.9 | 45.02 | 1093.6720 | 9 | -0.2 | 547.8431 | 2 | 21.66 |  |
| M.KNKVEIH.E | Y | 99.9 | 44.91 | 866.4974 | 7 | -0.5 | 434.2557 | 2 | 16.00 |  |
| A.VRGHPKPKVV.W | Y | 99.9 | 44.91 | 1115.6927 | 10 | -0.9 | 558.8531 | 2 | 22.60 |  |
| L.TREGSFK.S | Y | 99.9 | 44.90 | 823.4188 | 7 | 0.2 | 412.7167 | 2 | 10.77 |  |
| N.IAGRSEPA.T | Y | 99.9 | 44.88 | 799.4188 | 8 | -0.9 | 400.7163 | 2 | 19.90 |  |
| L.Q(-17.03)DIADLTVKAS.E | Y | 99.6 | 44.81 | 1142.5819 | 11 | -0.1 | 572.2982 | 2 | 103.25 | Pyro-glu from Q |
| E.EEKKKKKDDDDLGIPPEIW.E | Y | 99.9 | 44.76 | 2282.1794 | 19 | 0.6 | 571.5525 | 4 | 101.80 |  |
| F.KRAGDAKPD.G | Y | 99.9 | 44.63 | 956.5039 | 9 | -3.1 | 479.2578 | 2 | 5.52 |  |
| A.GPAENVM(+15.99)VK.E | Y | 99.9 | 44.61 | 959.4746 | 9 | -1.3 | 480.7440 | 2 | 54.07 | Oxidation (M) |
| K.VEYVPK.Q | Y | 99.9 | 44.56 | 733.4010 | 6 | 1.5 | 367.7083 | 2 | 37.65 |  |
| T.IKVTNPAGEDVA.T | Y | 99.9 | 44.52 | 1212.6350 | 12 | -0.5 | 607.3245 | 2 | 75.02 |  |
| K.KPDSVSVETGKDAVIM.A | Y | 99.6 | 44.46 | 1674.8499 | 16 | 2.8 | 838.4346 | 2 | 36.96 |  |
| K.ELPAKPT.I | Y | 99.9 | 44.29 | 754.4225 | 7 | -0.3 | 378.2184 | 2 | 55.31 |  |
| K.KREVVEEEKKKK.K | Y | 99.6 | 44.27 | 1528.8936 | 12 | -0.8 | 510.6380 | 3 | 16.23 |  |
| F.LKKPDSV.S | Y | 99.9 | 44.26 | 785.4647 | 7 | -0.3 | 393.7395 | 2 | 32.59 |  |
| T.IKVTNPAGEDVATVM(+15.99).L | Y | 99.9 | 44.26 | 1559.7865 | 15 | 0.1 | 780.9006 | 2 | 84.27 | Oxidation (M) |
| S.R(+27.99)VHVR.T | Y | 99.9 | 44.24 | 693.4034 | 5 | 1.5 | 347.7095 | 2 | 19.57 | Formylation |
| M.KDTATVR.I | Y | 99.9 | 44.24 | 789.4344 | 7 | -1.6 | 395.7239 | 2 | 5.79 |  |
| H.KLVIDDVRPEDEGDYT.F | Y | 99.9 | 44.18 | 1862.8898 | 16 | -0.6 | 621.9702 | 3 | 91.72 |  |
| Y.RFKKDGKRH.I | Y | 99.9 | 44.17 | 1170.6733 | 9 | -0.4 | 586.3437 | 2 | 18.21 |  |
| L.KTGITF.K | Y | 99.5 | 43.94 | 665.3748 | 6 | -2.3 | 333.6939 | 2 | 74.80 |  |
| R.GHPKPKVVW.M | Y | 99.9 | 43.82 | 1046.6025 | 9 | -1.6 | 349.8742 | 3 | 65.65 |  |
| Y.VFENVGKK.R | Y | 99.9 | 43.65 | 919.5127 | 8 | -1.4 | 460.7630 | 2 | 34.54 |  |
| L.EDRGHYQ.V | Y | 99.9 | 43.38 | 903.3835 | 7 | -3.6 | 452.6974 | 2 | 6.49 |  |
| F.NIDVEAPRQDSAGQSL.E | Y | 99.5 | 43.19 | 1698.8173 | 16 | 0.4 | 850.4163 | 2 | 86.65 |  |
| C.AVRGHPKPKVV.W | Y | 99.9 | 43.14 | 1186.7299 | 11 | -1.9 | 594.3711 | 2 | 28.26 |  |
| R.AFNELGEALAEC(+57.02)RLDVRVPQ | Y | 99.5 | 43.08 | 2286.1426 | 20 | 3.1 | 763.0571 | 3 | 117.35 | Carbamidomethylation |
| M.VKEVWGT.N | Y | 99.9 | 42.93 | 817.4334 | 7 | -0.3 | 409.7238 | 2 | 73.18 |  |
| C.VEGSDEWIPAN.K | Y | 99.9 | 42.82 | 1215.5408 | 11 | 0.7 | 608.7781 | 2 | 96.25 |  |
| T.GITFKPF.E | Y | 99.9 | 42.81 | 808.4483 | 7 | -0.6 | 405.2312 | 2 | 105.70 |  |
| A.ARSDSGEYELSVQ.I | Y | 99.9 | 42.35 | 1439.6528 | 13 | -0.4 | 720.8334 | 2 | 79.57 |  |
| K.NKVEIH.E | Y | 99.9 | 42.25 | 738.4024 | 6 | -0.9 | 370.2082 | 2 | 14.14 |  |
| K.KDGKRHIL.I | Y | 99.9 | 42.14 | 965.5770 | 8 | -0.7 | 483.7955 | 2 | 24.97 |  |
| T.SAPQHLTVE.D | Y | 99.5 | 42.14 | 980.4927 | 9 | -1.2 | 491.2530 | 2 | 33.75 |  |
| K.GKDAPKEEPPKEAAAEPPKEAPPEDQSPTAEEPTGIFLKKPDSVSVE.T | Y | 99.5 | 42.14 | 4966.4609 | 47 | -0.4 | 994.2991 | 5 | 95.00 |  |
| G.KVVLGDRGDYRLEVK.A | Y | 99.9 | 42.13 | 1745.9788 | 15 | -0.4 | 437.5018 | 4 | 80.23 |  |
| F.RSPPKFLTPL.I | Y | 99.9 | 41.78 | 1154.6812 | 10 | 0.0 | 578.3478 | 2 | 94.15 |  |
| K.GKDAPKEEPPKEAAAEPPKEAPPEDQSPTAEEPTGIFLKKPDSVSVETGKDAVIM.A | Y | 99.4 | 41.77 | 5781.8818 | 55 | 1.2 | 964.6554 | 6 | 34.37 |  |
| N.YRFKKDGKRHIL.I | Y | 99.9 | 41.72 | 1559.9048 | 12 | -1.4 | 780.9586 | 2 | 40.04 |  |
| L.KWRPPDRIGA.G | Y | 99.9 | 41.70 | 1194.6621 | 10 | -0.4 | 399.2278 | 3 | 71.61 |  |
| K.SNYRFKKD.G | Y | 99.9 | 41.66 | 1056.5352 | 8 | -1.1 | 529.2743 | 2 | 27.58 |  |
| E.WQPPKDNGNSE.I | Y | 99.9 | 41.49 | 1270.5579 | 11 | 0.4 | 636.2864 | 2 | 64.37 |  |
| A.GNKLRL.D | Y | 99.9 | 41.41 | 699.4391 | 6 | -0.7 | 350.7266 | 2 | 51.09 |  |
| W.MKGDEVF.T | Y | 99.9 | 41.35 | 824.3738 | 7 | -0.3 | 413.1941 | 2 | 39.01 |  |
| R.GDYRLE.V | Y | 99.9 | 41.23 | 751.3500 | 6 | 1.0 | 376.6827 | 2 | 61.23 |  |
| E.WQPPKDN.G | Y | 99.9 | 41.11 | 883.4188 | 7 | -0.5 | 442.7165 | 2 | 61.13 |  |
| C.AVRGHPKPK.V | Y | 99.9 | 41.01 | 988.5930 | 9 | -0.1 | 495.3037 | 2 | 11.39 |  |
| E.DRGHYQ.V | Y | 99.9 | 40.98 | 774.3409 | 6 | 0.1 | 388.1778 | 2 | 7.64 |  |
| K.Q(-17.03)LEVLQDIADLTVK.A | Y | 99.3 | 40.91 | 1566.8505 | 14 | -4.1 | 784.4293 | 2 | 34.83 | Pyro-glu from Q |
| T.SAPQHL.T | Y | 99.9 | 40.88 | 651.3340 | 6 | -0.1 | 326.6742 | 2 | 18.71 |  |
| M.NTKPFMPIAPT.S | Y | 99.9 | 40.88 | 1215.6322 | 11 | -0.5 | 608.8231 | 2 | 93.21 |  |
| F.ENVGKKRIL.T | Y | 99.9 | 40.80 | 1055.6451 | 9 | -1.5 | 528.8290 | 2 | 34.51 |  |
| K.VEIHEDPKFL.M | Y | 99.9 | 40.51 | 1225.6343 | 10 | 0.9 | 409.5524 | 3 | 89.57 |  |
| Q.VVWTK.G | Y | 99.9 | 40.45 | 631.3693 | 5 | -2.1 | 316.6913 | 2 | 81.91 |  |
| R.GDYRL.E | Y | 99.9 | 40.34 | 622.3074 | 5 | -0.8 | 312.1607 | 2 | 65.95 |  |
| F.FVREA.A | Y | 99.8 | 39.95 | 620.3282 | 5 | -1.5 | 311.1709 | 2 | 22.99 |  |
| T.GEPPPVAT.W | Y | 99.8 | 39.93 | 766.3861 | 8 | -2.5 | 384.1994 | 2 | 59.42 |  |
| L.F(+27.99)VKEPPVLIVTPL.E | Y | 99.8 | 39.89 | 1478.8748 | 13 | 1.4 | 740.4457 | 2 | 139.18 | Formylation |
| R.EIVEQPKIRLPRHLR.Q | Y | 99.8 | 39.79 | 1883.1217 | 15 | -0.2 | 471.7876 | 4 | 82.54 |  |
| T.WM(+15.99)KGDEVF.T | Y | 99.8 | 39.78 | 1026.4480 | 8 | -1.3 | 514.2306 | 2 | 85.33 | Oxidation (M) |
| V.GEHINL.V | Y | 99.8 | 39.71 | 681.3446 | 6 | -1.2 | 341.6792 | 2 | 70.18 |  |
| M.AKLNGKELPAKPTIK.W | Y | 99.0 | 39.71 | 1606.9769 | 15 | 1.2 | 402.7520 | 4 | 33.67 |  |
| L.ESFKRA.G | Y | 99.8 | 39.63 | 736.3867 | 6 | -0.5 | 369.2004 | 2 | 11.66 |  |
| N.IDVEAPRQDSAGQSLESFKR.A | Y | 99.0 | 39.43 | 2232.1133 | 20 | 0.0 | 559.0356 | 4 | 87.15 |  |
| E.Q(-17.03)RPDSSSFVIE.S | Y | 99.0 | 39.30 | 1246.5829 | 11 | 0.0 | 624.2987 | 2 | 99.79 | Pyro-glu from Q |
| C.EAELIVEEK.Q | Y | 99.8 | 39.29 | 1058.5496 | 9 | 0.7 | 530.2824 | 2 | 74.56 |  |
| H.EDPKFL.M | Y | 99.8 | 39.27 | 747.3802 | 6 | -0.8 | 374.6971 | 2 | 82.24 |  |
| Y.EHNRHT.S | Y | 99.8 | 38.84 | 792.3627 | 6 | 0.3 | 397.1887 | 2 | 3.87 |  |
| V.LGDRGDYR.L | Y | 99.8 | 38.78 | 950.4570 | 8 | -1.8 | 476.2349 | 2 | 33.28 |  |
| A.RSDSGEYELS.V | Y | 99.8 | 38.76 | 1141.4888 | 10 | -0.2 | 571.7515 | 2 | 64.90 |  |
| R.EIVEQPKIR.L | Y | 99.8 | 38.70 | 1110.6396 | 9 | -0.2 | 556.3270 | 2 | 67.88 |  |
| F.SFKETH.D | Y | 99.8 | 38.29 | 747.3551 | 6 | -4.4 | 374.6832 | 2 | 8.63 |  |
| L.NFLEIK.V | Y | 99.8 | 38.25 | 762.4276 | 6 | -0.5 | 382.2209 | 2 | 91.86 |  |
| W.M(+15.99)KGDEVFTVT.E | Y | 99.0 | 38.03 | 1141.5325 | 10 | -0.6 | 571.7732 | 2 | 79.59 | Oxidation (M) |
| R.KAPKGKDAPKEEPPKEAAAE.P | Y | 99.0 | 38.01 | 2090.1006 | 20 | -1.1 | 523.5319 | 4 | 33.41 |  |
| L.KKREVVEEE.K | Y | 99.8 | 37.89 | 1144.6088 | 9 | -3.6 | 573.3096 | 2 | 14.18 |  |
| R.KVGEHINLVIPFQGKPRPQVVWTK.G | Y | 99.0 | 37.86 | 2769.5806 | 24 | -2.4 | 554.9221 | 5 | 105.39 |  |
| F.VKEPPVLIV.T | Y | 99.7 | 37.25 | 992.6270 | 9 | -0.9 | 497.3203 | 2 | 35.00 |  |
| E.LIVEEK.Q | Y | 99.7 | 37.21 | 729.4272 | 6 | -1.0 | 365.7205 | 2 | 34.67 |  |
| Y.VRKVGEH.I | Y | 99.7 | 36.96 | 823.4664 | 7 | -4.2 | 412.7388 | 2 | 7.26 |  |
| F.LKKPDSVSVET.G | Y | 99.7 | 36.88 | 1201.6554 | 11 | 1.0 | 601.8356 | 2 | 63.26 |  |
| Q.TYVRK.V | Y | 99.7 | 36.87 | 665.3860 | 5 | -1.6 | 333.6998 | 2 | 8.29 |  |
| T.YVRKVGEHINL.V | Y | 99.7 | 36.82 | 1326.7407 | 11 | -0.7 | 664.3772 | 2 | 71.94 |  |
| S.LKYVF.E | Y | 99.7 | 36.80 | 668.3897 | 5 | -1.8 | 335.2015 | 2 | 91.57 |  |
| E.YKEHDFRSPPKFLTPL.I | Y | 99.7 | 36.69 | 1974.0363 | 16 | -0.1 | 494.5163 | 4 | 99.25 |  |
| R.SEPATLVQPVTIR.E | Y | 98.1 | 36.60 | 1409.7878 | 13 | 0.8 | 705.9017 | 2 | 93.61 |  |
| F.LKKPDSVSVETGKDAVIMA.K | Y | 98.1 | 36.58 | 1987.0659 | 19 | -0.1 | 663.3625 | 3 | 87.11 |  |
| W.M(+15.99)KGDEVF.T | Y | 99.7 | 36.39 | 840.3687 | 7 | -0.1 | 421.1916 | 2 | 60.87 | Oxidation (M) |
| R.KAPKGKDAPKEEPPKEAAAEPPKEAPPEDQSPTAEEPTGIFLKKPDSVSVETGKDAVIM(+15.99).A | Y | 98.1 | 36.30 | 6222.1567 | 59 | 2.0 | 778.7784 | 8 | 33.85 | Oxidation (M) |
| T.REGSFK.S | Y | 99.7 | 36.28 | 722.3711 | 6 | -1.6 | 362.1923 | 2 | 40.58 |  |
| S.NYRFK.K | Y | 99.7 | 36.22 | 726.3813 | 5 | -0.6 | 364.1977 | 2 | 34.48 |  |
| E.VNLEDR.G | Y | 99.6 | 36.00 | 744.3766 | 6 | -1.1 | 373.1952 | 2 | 34.61 |  |
| K.GKDAPKEEPPKEAAAEPPKEAPPEDQSPTAEEPTGIFLK.K | Y | 98.1 | 35.86 | 4125.0430 | 39 | 0.2 | 826.0161 | 5 | 92.37 |  |
| E.GSDEWIPA.N | Y | 99.6 | 35.81 | 873.3868 | 8 | -0.7 | 437.7004 | 2 | 95.67 |  |
| T.AEEPTGIF.L | Y | 99.6 | 35.34 | 862.4072 | 8 | -1.1 | 432.2104 | 2 | 99.84 |  |
| L.KKREVVEE.E | Y | 99.6 | 35.20 | 1015.5662 | 8 | 0.7 | 508.7907 | 2 | 9.31 |  |
| K.GLPTGAK.I | Y | 97.9 | 35.17 | 642.3701 | 7 | -0.8 | 322.1920 | 2 | 25.29 |  |
| C.VEGSDEWIPA.N | Y | 99.6 | 34.86 | 1101.4978 | 10 | 1.2 | 551.7568 | 2 | 156.02 |  |
| R.KVGEHINLVIPF.Q | Y | 97.8 | 34.75 | 1364.7816 | 12 | -2.7 | 683.3962 | 2 | 34.15 |  |
| E.NM(+15.99)KDTATVR.I | Y | 99.5 | 34.72 | 1050.5128 | 9 | -1.2 | 526.2631 | 2 | 7.39 | Oxidation (M) |
| T.REGSFKS.N | Y | 99.5 | 34.64 | 809.4031 | 7 | 0.0 | 405.7088 | 2 | 8.15 |  |
| R.IEQRPDSSSFVIE.S | Y | 96.7 | 34.26 | 1505.7361 | 13 | 2.3 | 753.8771 | 2 | 91.46 |  |
| S.FKETHDA.A | Y | 99.5 | 34.20 | 846.3871 | 7 | -2.2 | 424.1999 | 2 | 7.58 |  |
| L.FVKEPPVLI.V | Y | 99.5 | 34.20 | 1040.6270 | 9 | -1.2 | 521.3201 | 2 | 35.00 |  |
| A.RSDSGEYEL.S | Y | 99.5 | 34.02 | 1054.4567 | 9 | 1.8 | 528.2366 | 2 | 72.95 |  |
| F.KRAGDAKPDGAGELDFSGLLKKREVVEEEKKK.K | Y | 96.7 | 33.76 | 3526.9106 | 32 | 0.9 | 588.8262 | 6 | 91.01 |  |
| L.VEWQPPKDNGNSE.I | Y | 99.5 | 33.73 | 1498.6688 | 13 | -0.6 | 750.3412 | 2 | 75.59 |  |
| E.DEGDYTFVPDGYAL.S | Y | 99.5 | 33.71 | 1560.6620 | 14 | -2.1 | 781.3366 | 2 | 121.45 |  |
| S.EQAM(+15.99)FK.C | Y | 96.6 | 33.63 | 768.3476 | 6 | -1.5 | 385.1805 | 2 | 18.96 | Oxidation (M) |
| T.NALVEWQPPKDNGNSEITGY.L | Y | 96.6 | 33.44 | 2231.0493 | 20 | 1.9 | 744.6918 | 3 | 108.44 |  |
| G.KVVLGDRGDYRLE.V | Y | 99.4 | 33.44 | 1518.8154 | 13 | -0.7 | 507.2787 | 3 | 78.41 |  |
| D.DVRPEDEGDYTFVPDGYAL.S | Y | 99.4 | 33.31 | 2156.9539 | 19 | -0.4 | 1079.4838 | 2 | 117.66 |  |
| R.KAPKGKDAPKEEPPKEAAAEPPKEAPPEDQSPTAEEPTGIFLKKPDSVSVETGKDAVIM.A | Y | 96.5 | 33.20 | 6206.1616 | 59 | -3.8 | 887.5984 | 7 | 100.59 |  |
| N.FLEIK.V | Y | 99.4 | 33.09 | 648.3846 | 5 | -1.1 | 325.1992 | 2 | 35.98 |  |
| I.SDPDLPLK.W | Y | 99.4 | 33.08 | 883.4651 | 8 | -0.9 | 442.7394 | 2 | 75.44 |  |
| L.LKKREVVE.E | Y | 99.4 | 32.85 | 999.6077 | 8 | -0.5 | 500.8109 | 2 | 19.41 |  |
| Q.VDRGNKVK.L | Y | 99.4 | 32.39 | 914.5297 | 8 | -0.4 | 458.2720 | 2 | 6.92 |  |
| T.KKLDPA.Y | Y | 99.4 | 32.34 | 670.4014 | 6 | -0.4 | 336.2078 | 2 | 8.31 |  |
| E.Q(-17.03)PKIRLPRHL.R | Y | 99.4 | 32.34 | 1239.7563 | 10 | -4.3 | 414.2576 | 3 | 91.26 | Pyro-glu from Q |
| V.VEISDPDLPL.K | Y | 99.3 | 32.31 | 1096.5652 | 10 | -0.8 | 549.2894 | 2 | 111.40 |  |
| Y.GITDLR.G | N | 99.3 | 32.12 | 673.3759 | 6 | -0.2 | 337.6951 | 2 | 66.96 |  |
| Y.VFENVGKKRIL.T | Y | 99.3 | 32.04 | 1301.7819 | 11 | -0.5 | 434.9343 | 3 | 75.94 |  |
| E.ISDPDLPL.K | Y | 99.3 | 32.02 | 868.4542 | 8 | 0.3 | 435.2345 | 2 | 103.09 |  |
| E.VSEEGAQVM(+15.99).W | Y | 99.2 | 31.87 | 964.4172 | 9 | -4.6 | 483.2137 | 2 | 11.27 | Oxidation (M) |
| Y.LLERK.K | Y | 99.2 | 31.78 | 657.4174 | 5 | -0.4 | 329.7158 | 2 | 18.93 |  |
| T.WMKGD.E | Y | 99.2 | 31.68 | 635.2737 | 5 | -1.5 | 318.6437 | 2 | 32.69 |  |
| K.GLPTGAKIL.F | Y | 99.2 | 31.67 | 868.5381 | 9 | -0.6 | 435.2761 | 2 | 92.19 |  |
| E.VRPSKRIT.I | Y | 99.2 | 31.66 | 955.5927 | 8 | -0.5 | 478.8033 | 2 | 16.64 |  |
| L.LKKREVVEEEK.K | Y | 96.0 | 31.58 | 1385.7877 | 11 | 2.2 | 693.9026 | 2 | 19.63 |  |
| V.EEEKKKKKDDDDLGIPPEIW.E | Y | 99.1 | 31.22 | 2411.2219 | 20 | 1.8 | 603.8138 | 4 | 100.93 |  |
| L.LERKKKGSQR.W | Y | 99.0 | 31.19 | 1228.7363 | 10 | -0.6 | 410.5858 | 3 | 16.85 |  |
| L.FRVIGV.N | Y | 98.9 | 30.76 | 689.4224 | 6 | -1.3 | 345.7180 | 2 | 34.96 |  |
| R.EIVEQPKIRLPRHLRQT.Y | Y | 98.9 | 30.63 | 2112.2280 | 17 | 0.1 | 529.0643 | 4 | 82.70 |  |
| R.AFNELGEALAE.C | Y | 92.5 | 30.33 | 1162.5505 | 11 | -1.1 | 582.2819 | 2 | 106.82 |  |
| S.KRITIS.H | Y | 98.7 | 30.26 | 716.4545 | 6 | -0.5 | 359.2343 | 2 | 25.78 |  |
| K.EAPPEDQSPTAEEPTGIFLKKPDSVSVETGKDAVIM.A | Y | 92.4 | 30.16 | 3811.8713 | 36 | 0.4 | 953.9755 | 4 | 110.15 |  |
| F.KNGQEIK.P | Y | 98.6 | 30.04 | 815.4501 | 7 | 4.3 | 408.7341 | 2 | 96.90 |  |
| K.DAPKEEPPKEAAAEPPKEAPPEDQSPTAEEPTGIF.L | Y | 92.3 | 30.02 | 3698.7473 | 35 | -1.3 | 925.6929 | 4 | 96.01 |  |
| A.KVEVK.K | Y | 98.6 | 29.85 | 601.3799 | 5 | -2.1 | 301.6966 | 2 | 6.71 |  |
| Y.Q(-17.03)VDRGNKVK.L | Y | 98.6 | 29.83 | 1025.5618 | 9 | -0.6 | 513.7878 | 2 | 12.77 | Pyro-glu from Q |
| N.KVEIH.E | Y | 98.5 | 29.61 | 624.3595 | 5 | -0.4 | 313.1869 | 2 | 12.24 |  |
| E.QRPDSSSFVIE.S | Y | 91.9 | 29.48 | 1263.6095 | 11 | -0.8 | 632.8115 | 2 | 84.51 |  |
| R.VRIEQRPDSSSFVIE.S | Y | 91.8 | 29.31 | 1760.9056 | 15 | 0.2 | 587.9760 | 3 | 91.51 |  |
| K.LVVEISDPDLPLKWFKNGQEIKPS.L | Y | 98.4 | 29.06 | 2751.4846 | 24 | 2.6 | 918.1712 | 3 | 121.85 |  |
| N.VGKKRIL.T | Y | 98.4 | 28.98 | 812.5596 | 7 | -0.3 | 407.2869 | 2 | 25.10 |  |
| S.GLLKKR.E | Y | 98.3 | 28.95 | 713.4911 | 6 | -1.3 | 357.7524 | 2 | 18.08 |  |
| R.QTYVR.K | Y | 91.7 | 28.90 | 665.3497 | 5 | 3.2 | 333.6832 | 2 | 18.52 |  |
| Q.VDRGNKV.K | Y | 98.3 | 28.72 | 786.4348 | 7 | -1.3 | 394.2242 | 2 | 6.36 |  |
| D.GKRHIL.I | Y | 98.3 | 28.58 | 722.4551 | 6 | 0.6 | 362.2350 | 2 | 23.95 |  |
| L.LKKREVVEEEKKKKK.D | Y | 98.3 | 28.42 | 1898.1676 | 15 | -0.5 | 633.7295 | 3 | 33.44 |  |
| G.K(+27.99)DAPK(+27.99)EEPPKEAAAEPPKEAPPEDQSPTAEEPTGIFLKKPDSVSVETGKDAVIM(+15.99).A | Y | 91.5 | 28.24 | 5796.8452 | 54 | 2.9 | 967.1509 | 6 | 101.46 | Formylation; Oxidation (M) |
| F.EYKEHDFRSPPKFL.T | Y | 97.7 | 27.98 | 1791.8943 | 14 | 0.1 | 598.3054 | 3 | 87.91 |  |
| Y.LLERKK.K | Y | 91.3 | 27.64 | 785.5123 | 6 | -0.9 | 393.7631 | 2 | 11.90 |  |
| M.KGDEVF.T | Y | 97.5 | 27.57 | 693.3333 | 6 | -1.9 | 347.6733 | 2 | 63.65 |  |
| V.KEPPVLIVTPL.E | Y | 97.5 | 27.45 | 1204.7430 | 11 | 0.2 | 603.3789 | 2 | 111.29 |  |
| R.EAARSDSGEYELSVQ.I | Y | 97.5 | 27.38 | 1639.7325 | 15 | -0.2 | 820.8734 | 2 | 81.64 |  |
| Q.SLESFKRAGDAKPDGAGELDFSGLLK.K | Y | 91.2 | 27.30 | 2707.3816 | 26 | 0.6 | 677.8531 | 4 | 108.74 |  |
| S.RVHVR.T | Y | 97.5 | 27.26 | 665.4085 | 5 | -0.7 | 333.7113 | 2 | 17.93 |  |
| A.AAEPPKEAPPEDQSPTAEEPTGIFLKKPDSVSVETGKDAVIM.A | Y | 91.1 | 27.26 | 4405.1885 | 42 | 0.9 | 882.0458 | 5 | 107.46 |  |
| V.DVPDPPEAVRVT.S | Y | 97.4 | 27.20 | 1293.6565 | 12 | 1.4 | 647.8364 | 2 | 84.92 |  |
| A.ENVM(+15.99)VK.E | Y | 97.4 | 27.18 | 734.3633 | 6 | -0.6 | 368.1887 | 2 | 8.21 | Oxidation (M) |
| L.EDQQVF.V | Y | 97.4 | 27.12 | 764.3340 | 6 | 0.2 | 383.1744 | 2 | 76.50 |  |
| R.VHVRTS.D | Y | 91.1 | 27.10 | 697.3871 | 6 | -2.1 | 349.7001 | 2 | 11.08 |  |
| Q.SLESFKRAGDAKPDGAGELDFSGL.L | Y | 90.8 | 26.90 | 2466.2026 | 24 | 0.4 | 617.5582 | 4 | 106.17 |  |
| T.EGRVR.I | Y | 96.7 | 26.41 | 615.3452 | 5 | 0.4 | 308.6800 | 2 | 7.15 |  |
| V.GKKRIL.T | Y | 96.7 | 26.37 | 713.4911 | 6 | -0.5 | 357.7527 | 2 | 19.88 |  |
| F.LKKPDS.V | Y | 96.6 | 26.21 | 686.3962 | 6 | -2.5 | 344.2045 | 2 | 31.88 |  |
| S.LKYVFE.N | Y | 96.6 | 26.17 | 797.4323 | 6 | -0.8 | 399.7231 | 2 | 89.14 |  |
| A.TVRIR.V | Y | 96.6 | 26.11 | 643.4129 | 5 | -0.4 | 322.7136 | 2 | 20.74 |  |
| V.EQPKIRLPRHL.R | Y | 96.6 | 26.04 | 1385.8254 | 11 | -2.5 | 347.4628 | 4 | 79.19 |  |
| R.KAPKGKDAPKEEPPKEAAAEPPKEAPPEDQSPTAEEPTGIFLKKPDSVSVE.T | Y | 82.7 | 25.60 | 5390.7407 | 51 | -1.4 | 899.4628 | 6 | 91.95 |  |
| E.GSDEWIPAN.K | Y | 96.2 | 25.40 | 987.4297 | 9 | 0.4 | 494.7224 | 2 | 93.05 |  |
| R.KAPKGKDAPKEEPPKEAAAEPPKEAPPEDQSPTAEEPTGIF.L | Y | 81.7 | 25.12 | 4308.1436 | 41 | 1.7 | 862.6375 | 5 | 38.46 |  |
| K.WFKGK.W | Y | 95.6 | 25.07 | 664.3696 | 5 | -3.1 | 333.1911 | 2 | 51.83 |  |
| E.LIVEEKQLEVL.Q | Y | 81.6 | 25.06 | 1311.7649 | 11 | -0.3 | 656.8895 | 2 | 107.83 |  |
| T.IKVTNPAGEDVATV.M | Y | 95.5 | 25.02 | 1412.7511 | 14 | 0.1 | 707.3829 | 2 | 87.42 |  |
| A.AAEPPKEAPPEDQSPTAEEPTGIFLKKPDSVSVETGKDAVIM(+15.99).A | Y | 81.2 | 24.66 | 4421.1836 | 42 | 3.2 | 885.2468 | 5 | 102.47 | Oxidation (M) |
| L.RGMLK.R | N | 95.2 | 24.55 | 603.3527 | 5 | -1.7 | 302.6831 | 2 | 19.41 |  |
| K.VVLGDRGDYRL.E | Y | 95.0 | 24.26 | 1261.6779 | 11 | -0.4 | 421.5664 | 3 | 87.29 |  |
| D.APKEEPPKEAA.A | Y | 95.0 | 24.26 | 1165.5978 | 11 | -0.6 | 583.8058 | 2 | 30.05 |  |
| E.APPEDQSPTAEEPTGIF.L | Y | 95.0 | 24.23 | 1784.8104 | 17 | 0.5 | 893.4129 | 2 | 104.44 |  |
| A.AEPPKEAPPEDQSPTAEEPTGIFL.K | Y | 95.0 | 24.18 | 2549.2173 | 24 | 0.4 | 850.7467 | 3 | 109.91 |  |
| E.KQLEVL.Q | Y | 95.0 | 24.12 | 728.4432 | 6 | -3.2 | 365.2277 | 2 | 75.67 |  |
| K.DAPKEEPPKEAAAEPPKEAPPEDQSPTAEEPTGIFLKKPDSVSVETGKDAVIM(+15.99).A | Y | 80.4 | 24.01 | 5612.7607 | 53 | -3.1 | 1123.5559 | 5 | 100.72 | Oxidation (M) |
| T.LKWRPPDRIGAG.G | Y | 94.7 | 23.92 | 1364.7677 | 12 | -1.0 | 455.9294 | 3 | 82.77 |  |
| K.WRPPDRIGAG.G | Y | 93.9 | 23.77 | 1123.5886 | 10 | 0.7 | 562.8020 | 2 | 38.74 |  |
| G.DAKPDGAGEL.D | Y | 93.9 | 23.74 | 971.4559 | 10 | -0.7 | 486.7349 | 2 | 61.67 |  |
| L.KKPDSVSVETGKDAVIMA.K | Y | 80.0 | 23.72 | 1873.9818 | 18 | -0.6 | 625.6675 | 3 | 81.05 |  |
| Y.LLERKKK.G | Y | 79.9 | 23.58 | 913.6072 | 7 | -2.2 | 457.8099 | 2 | 11.21 |  |
| Q.VDRGNKVKLVVEISDPDLPLKWFKNGQ.E | Y | 93.7 | 23.58 | 3093.6975 | 27 | 1.4 | 774.4327 | 4 | 118.17 |  |
| A.PKGKDAPKEEPPKEA.A | Y | 93.7 | 23.53 | 1619.8518 | 15 | -0.9 | 540.9574 | 3 | 34.30 |  |
| F.EYKEHDFRSPPKFLTPLIDRVVVA.G | Y | 93.6 | 23.31 | 2855.5332 | 24 | -0.4 | 714.8903 | 4 | 116.78 |  |
| K.EAPPEDQSPTAEEPTGIFL.K | Y | 92.8 | 23.10 | 2026.9370 | 19 | 0.8 | 1014.4766 | 2 | 115.86 |  |
| A.LVEWQPPKDNGNSE.I | Y | 92.4 | 22.91 | 1611.7528 | 14 | -1.2 | 806.8827 | 2 | 86.95 |  |
| M.LKRLK.K | N | 92.4 | 22.87 | 656.4697 | 5 | -0.7 | 329.2419 | 2 | 13.97 |  |
| H.INLVIPF.Q | Y | 92.4 | 22.83 | 814.4952 | 7 | 1.0 | 408.2553 | 2 | 131.72 |  |
| L.TLNIRRPSPF.D | Y | 92.3 | 22.72 | 1199.6775 | 10 | -0.1 | 600.8459 | 2 | 88.53 |  |
| G.KVVLGDRGDYR.L | Y | 92.3 | 22.49 | 1276.6887 | 11 | -0.6 | 426.5699 | 3 | 58.63 |  |
| L.VWDPPKYDGGQPVTG.Y | Y | 92.2 | 22.46 | 1614.7678 | 15 | 2.7 | 808.3934 | 2 | 90.38 |  |
| S.HVGRFHKL.V | Y | 89.5 | 22.24 | 992.5668 | 8 | -0.2 | 331.8628 | 3 | 34.45 |  |
| Y.RFKKD.G | Y | 88.7 | 21.99 | 692.3969 | 5 | -2.6 | 347.2048 | 2 | 6.27 |  |
| L.FVKEPPVLIVTPLEDQQVFVGD.R | Y | 88.5 | 21.86 | 2468.3203 | 22 | -0.7 | 823.7802 | 3 | 128.65 |  |
| A.YQVDRGNKVKLVVEISDPDLPLKWFKNGQEIKPS.L | Y | 87.8 | 21.46 | 3939.1257 | 34 | -2.0 | 985.7867 | 4 | 117.40 |  |
| A.GRSEPATLVQPVTIREIVEQPKIRLPR.H | Y | 59.9 | 21.26 | 3081.7773 | 27 | 1.4 | 617.3636 | 5 | 111.04 |  |
| K.REVVEEEK.K | Y | 59.7 | 21.06 | 1016.5138 | 8 | 0.3 | 509.2643 | 2 | 14.02 |  |
| N.IRRPSPF.D | Y | 87.4 | 21.02 | 871.5028 | 7 | -0.9 | 436.7583 | 2 | 67.77 |  |
| K.GKDAPKEEPPKEAAAEPPKEAPPEDQSPTAEEPTGIFLKKPDSVSVETGK.D | Y | 59.6 | 20.98 | 5252.6250 | 50 | 0.1 | 1051.5323 | 5 | 91.80 |  |
| K.KGSQRW.M | Y | 87.0 | 20.65 | 760.3980 | 6 | 2.3 | 381.2072 | 2 | 22.68 |  |
| F.KSNYRFK.K | Y | 59.1 | 20.49 | 941.5082 | 7 | -2.1 | 471.7604 | 2 | 21.31 |  |
| M.LRVVDVPD.P | Y | 86.8 | 20.46 | 911.5076 | 8 | -1.3 | 456.7605 | 2 | 83.79 |  |
| M.W(+27.99)MKDGVELT.R | Y | 81.1 | 20.26 | 1105.5114 | 9 | 0.9 | 553.7634 | 2 | 86.55 | Formylation |
| K.DAPKEEPPKEAAAEPPKEAPPEDQSPTAEEPTGIFLKKPDSVSVETGKDAVIM.A | Y | 58.6 | 19.90 | 5596.7656 | 53 | 1.3 | 933.8027 | 6 | 104.94 |  |
| E.NVGKKRIL.T | Y | 80.2 | 19.14 | 926.6025 | 8 | -0.6 | 309.8746 | 3 | 29.23 |  |
| L.KKREVVEEEKKK.K | Y | 57.8 | 19.03 | 1528.8936 | 12 | 0.5 | 765.4544 | 2 | 16.12 |  |
| F.V(+27.99)K(+27.99)EPPVLIVTPL.E | Y | 79.6 | 18.84 | 1359.8013 | 12 | 2.9 | 680.9099 | 2 | 125.47 | Formylation |
| R.KAPKGKDAPKEEPPKEAAAEPPKEAPPEDQSPTAEEPTGIFLK.K | Y | 57.3 | 18.55 | 4549.3228 | 43 | 0.3 | 910.8721 | 5 | 87.94 |  |
| G.RFHKL.V | Y | 74.6 | 17.92 | 699.4180 | 5 | -2.4 | 350.7154 | 2 | 23.00 |  |
| V.FFVRE.A | Y | 74.5 | 17.87 | 696.3595 | 5 | -0.9 | 349.1867 | 2 | 76.13 |  |
| K.KPDSVSVETGKDAVIM(+15.99).A | Y | 49.6 | 17.60 | 1690.8447 | 16 | 0.0 | 846.4296 | 2 | 74.62 | Oxidation (M) |
| L.VVEISDPDLPL.K | Y | 73.1 | 17.56 | 1195.6335 | 11 | 0.1 | 598.8241 | 2 | 114.97 |  |
| A.ASNVYSLELHI.G | Y | 73.0 | 17.53 | 1244.6401 | 11 | -3.0 | 623.3255 | 2 | 20.83 |  |
| V.TIREIVEQPKIRLPR.H | Y | 69.7 | 17.31 | 1847.1105 | 15 | -2.0 | 616.7095 | 3 | 91.39 |  |
| E.S(+27.99)FK(+27.99)RAGD.A | Y | 69.5 | 17.20 | 835.3824 | 7 | -2.8 | 418.6973 | 2 | 9.47 | Formylation |
| L.TVEDVTDTTTTL.K | Y | 48.7 | 17.08 | 1294.6140 | 12 | -1.0 | 648.3137 | 2 | 90.85 |  |
| N.GVEVRPSKRITIS.H | Y | 69.2 | 17.00 | 1440.8412 | 13 | -1.4 | 481.2870 | 3 | 69.33 |  |
| R.EVVEEEKK(+27.99)KKK.D | Y | 48.6 | 17.00 | 1400.7875 | 11 | 2.3 | 351.2050 | 4 | 19.45 | Formylation |
| P.EAK(+27.99)PAARK.A | Y | 69.0 | 16.82 | 897.5032 | 8 | 0.5 | 449.7591 | 2 | 7.73 | Formylation |
| L.PAKPTIK.W | Y | 68.9 | 16.78 | 753.4749 | 7 | -0.6 | 377.7445 | 2 | 63.49 |  |
| L.KWRPPDRIGAGGID.G | Y | 64.0 | 15.88 | 1536.8160 | 14 | -0.2 | 513.2792 | 3 | 84.02 |  |
| K.SNYRFKKDGKRH.I | Y | 50.8 | 15.73 | 1534.8116 | 12 | -3.1 | 384.7090 | 4 | 34.03 |  |
| K.REVVEEEKK.K | Y | 46.2 | 15.65 | 1144.6088 | 9 | -3.2 | 573.3098 | 2 | 11.38 |  |
| A.E(+27.99)CRLDVR.V | Y | 46.1 | 15.63 | 917.4389 | 7 | -0.5 | 459.7265 | 2 | 28.39 | Formylation |
| D.APKEEPPKEAAAEPPKEAPPEDQSPTAEEPTGIFLKKPDSVSVETGKDAVIM.A | Y | 45.4 | 15.25 | 5481.7388 | 52 | -0.2 | 914.6302 | 6 | 104.00 |  |
| **total 531 peptides** |  |  |  |  |  |  |  |  |  |  |

The file that presents expanded data on MyBP-C mass spectrometry is here:

<https://drive.google.com/file/d/1CYWtyUrlNMoUywsyDHtr-jweFvVb_j2G/view?usp=sharing>

**Myosin binding protein-C forms amyloid-like aggregates *in vitro***

*Liya Bobyleva^1^, Sergey Shumeyko^1^, Elmira Yakupova^1^, Alexey Surin^2,3,4^, Oxana Galzitskaya^1,2^, Hiroshi Kihara^5^, Alexander Timchenko^6^, Maria Timchenko^7^, Nikita Penkov^8^, Alexey Nikulin^9^, Mariya Suvorina^2^, Nikolay Molochkov^10^, Mikhail Lobanov^2^, Roman Fadeev^11^, Ivan Vikhlyantsev^1,^* & Alexander Bobylev^1,^**

^1^Laboratory of the Structure and Functions of Muscle Proteins, Institute of Theoretical and Experimental Biophysics, Russian Academy of Sciences, Pushchino, Moscow Region 142290, Russia; ^2^Laboratory of Bioinformatics and Proteomics, Institute of Protein Research, Russian Academy of Sciences, Pushchino, Moscow Region 142290, Russia; ^3^Biological Testing Laboratory, Branch of the Shemyakin–Ovchinnikov Institute of Bioorganic Chemistry, Russian Academy of Sciences, Pushchino, Moscow Region 142290, Russia; ^4^Laboratory of the Biochemistry of Pathogenic Microorganisms, State Research Centre for Applied Microbiology and Biotechnology, Obolensk, Serpukhov District, Moscow Region, 142279, Russia; ^5^Department of Early Childhood Education, Himeji-Hinomoto College, 890 Koro, Kodera-cho, Himeji 679-2151, Japan; ^6^Group of Experimental Research and Engineering of Oligomeric Structures, Institute of Protein Research, Russian Academy of Sciences, Pushchino, Moscow Region 142290, Russia; ^7^Laboratory of Applied Enzymology, FRC PSCBR, Russian Academy of Sciences, Pushchino, Moscow Region 142290, Russia; ^8^Laboratory of the Methods of Optical Spectral Analysis, Institute of Cell Biophysics, Russian Academy of Sciences, FRC PSCBR RAS, Pushchino, Moscow Region 142290, Russia; ^9^Laboratory for Structural Studies of the Translational Apparatus, Institute of Protein Research, Russian Academy of Sciences, Pushchino, Moscow Region 142290, Russia; ^10^Laboratory of NMR Investigations of Biosystems, Institute of Theoretical and Experimental Biophysics, Russian Academy of Sciences, Pushchino, Moscow Region 142290, Russia; ^11^Laboratory of Pharmacological Regulation of Cell Resistance, Institute of Theoretical and Experimental Biophysics, Russian Academy of Sciences, Pushchino, Moscow Region 142290, Russia.


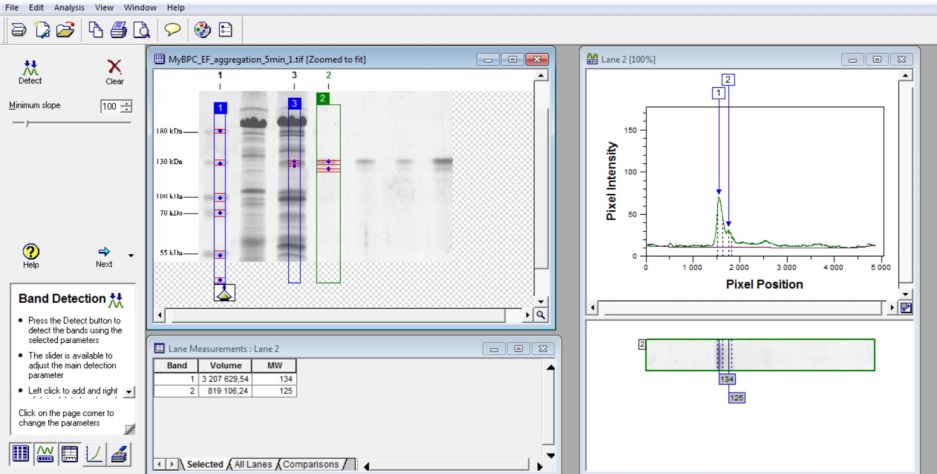


Supplementary Fig. S1. Determination of the molecular weight of sMyBP-C using Total Lab V 1.11 software.

The original image is here:

<https://drive.google.com/file/d/1THBQ23ezEtVeIx3_WKw6Py2Ql0ixHUVH/view?usp=sharing>

**Myosin binding protein-C forms amyloid-like aggregates *in vitro***

*Liya Bobyleva^1^, Sergey Shumeyko^1^, Elmira Yakupova^1^, Alexey Surin^2,3,4^, Oxana Galzitskaya^1,2^, Hiroshi Kihara^5^, Alexander Timchenko^6^, Maria Timchenko^7^, Nikita Penkov^8^, Alexey Nikulin^9^, Mariya Suvorina^2^, Nikolay Molochkov^10^, Mikhail Lobanov^2^, Roman Fadeev^11^, Ivan Vikhlyantsev^1,^* & Alexander Bobylev^1,^**

^1^Laboratory of the Structure and Functions of Muscle Proteins, Institute of Theoretical and Experimental Biophysics, Russian Academy of Sciences, Pushchino, Moscow Region 142290, Russia; ^2^Laboratory of Bioinformatics and Proteomics, Institute of Protein Research, Russian Academy of Sciences, Pushchino, Moscow Region 142290, Russia; ^3^Biological Testing Laboratory, Branch of the Shemyakin–Ovchinnikov Institute of Bioorganic Chemistry, Russian Academy of Sciences, Pushchino, Moscow Region 142290, Russia; ^4^Laboratory of the Biochemistry of Pathogenic Microorganisms, State Research Centre for Applied Microbiology and Biotechnology, Obolensk, Serpukhov District, Moscow Region, 142279, Russia; ^5^Department of Early Childhood Education, Himeji-Hinomoto College, 890 Koro, Kodera-cho, Himeji 679-2151, Japan; ^6^Group of Experimental Research and Engineering of Oligomeric Structures, Institute of Protein Research, Russian Academy of Sciences, Pushchino, Moscow Region 142290, Russia; ^7^Laboratory of Applied Enzymology, FRC PSCBR, Russian Academy of Sciences, Pushchino, Moscow Region 142290, Russia; ^8^Laboratory of the Methods of Optical Spectral Analysis, Institute of Cell Biophysics, Russian Academy of Sciences, FRC PSCBR RAS, Pushchino, Moscow Region 142290, Russia; ^9^Laboratory for Structural Studies of the Translational Apparatus, Institute of Protein Research, Russian Academy of Sciences, Pushchino, Moscow Region 142290, Russia; ^10^Laboratory of NMR Investigations of Biosystems, Institute of Theoretical and Experimental Biophysics, Russian Academy of Sciences, Pushchino, Moscow Region 142290, Russia; ^11^Laboratory of Pharmacological Regulation of Cell Resistance, Institute of Theoretical and Experimental Biophysics, Russian Academy of Sciences, Pushchino, Moscow Region 142290, Russia.


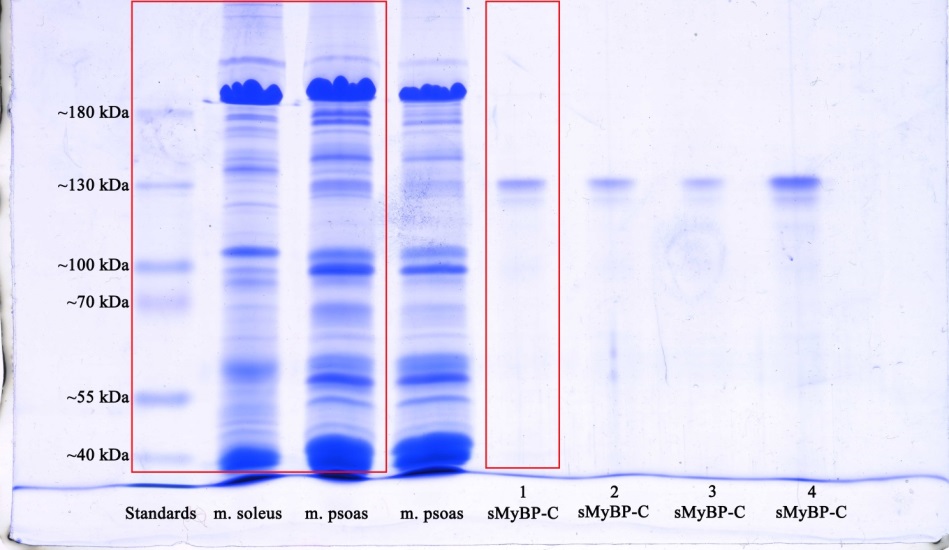


Supplementary Fig. S2. Full-length gel with designated segments (red boxes) used for Fig. 2.

The following specimens (left to right) are present on the gel: molecular weight standards, rabbit m. soleus samples, rabbit m. psoas in two repeats, isolated sMyBP-C protein (1–4) applied at different concentrations.

The original image is here:

<https://drive.google.com/file/d/1THBQ23ezEtVeIx3_WKw6Py2Ql0ixHUVH/view?usp=sharing>

**Myosin binding protein-C forms amyloid-like aggregates *in vitro***

*Liya Bobyleva^1^, Sergey Shumeyko^1^, Elmira Yakupova^1^, Alexey Surin^2,3,4^, Oxana Galzitskaya^1,2^, Hiroshi Kihara^5^, Alexander Timchenko^6^, Maria Timchenko^7^, Nikita Penkov^8^, Alexey Nikulin^9^, Mariya Suvorina^2^, Nikolay Molochkov^10^, Mikhail Lobanov^2^, Roman Fadeev^11^, Ivan Vikhlyantsev^1,^* & Alexander Bobylev^1,^**

^1^Laboratory of the Structure and Functions of Muscle Proteins, Institute of Theoretical and Experimental Biophysics, Russian Academy of Sciences, Pushchino, Moscow Region 142290, Russia; ^2^Laboratory of Bioinformatics and Proteomics, Institute of Protein Research, Russian Academy of Sciences, Pushchino, Moscow Region 142290, Russia; ^3^Biological Testing Laboratory, Branch of the Shemyakin–Ovchinnikov Institute of Bioorganic Chemistry, Russian Academy of Sciences, Pushchino, Moscow Region 142290, Russia; ^4^Laboratory of the Biochemistry of Pathogenic Microorganisms, State Research Centre for Applied Microbiology and Biotechnology, Obolensk, Serpukhov District, Moscow Region, 142279, Russia; ^5^Department of Early Childhood Education, Himeji-Hinomoto College, 890 Koro, Kodera-cho, Himeji 679-2151, Japan; ^6^Group of Experimental Research and Engineering of Oligomeric Structures, Institute of Protein Research, Russian Academy of Sciences, Pushchino, Moscow Region 142290, Russia; ^7^Laboratory of Applied Enzymology, FRC PSCBR, Russian Academy of Sciences, Pushchino, Moscow Region 142290, Russia; ^8^Laboratory of the Methods of Optical Spectral Analysis, Institute of Cell Biophysics, Russian Academy of Sciences, FRC PSCBR RAS, Pushchino, Moscow Region 142290, Russia; ^9^Laboratory for Structural Studies of the Translational Apparatus, Institute of Protein Research, Russian Academy of Sciences, Pushchino, Moscow Region 142290, Russia; ^10^Laboratory of NMR Investigations of Biosystems, Institute of Theoretical and Experimental Biophysics, Russian Academy of Sciences, Pushchino, Moscow Region 142290, Russia; ^11^Laboratory of Pharmacological Regulation of Cell Resistance, Institute of Theoretical and Experimental Biophysics, Russian Academy of Sciences, Pushchino, Moscow Region 142290, Russia.


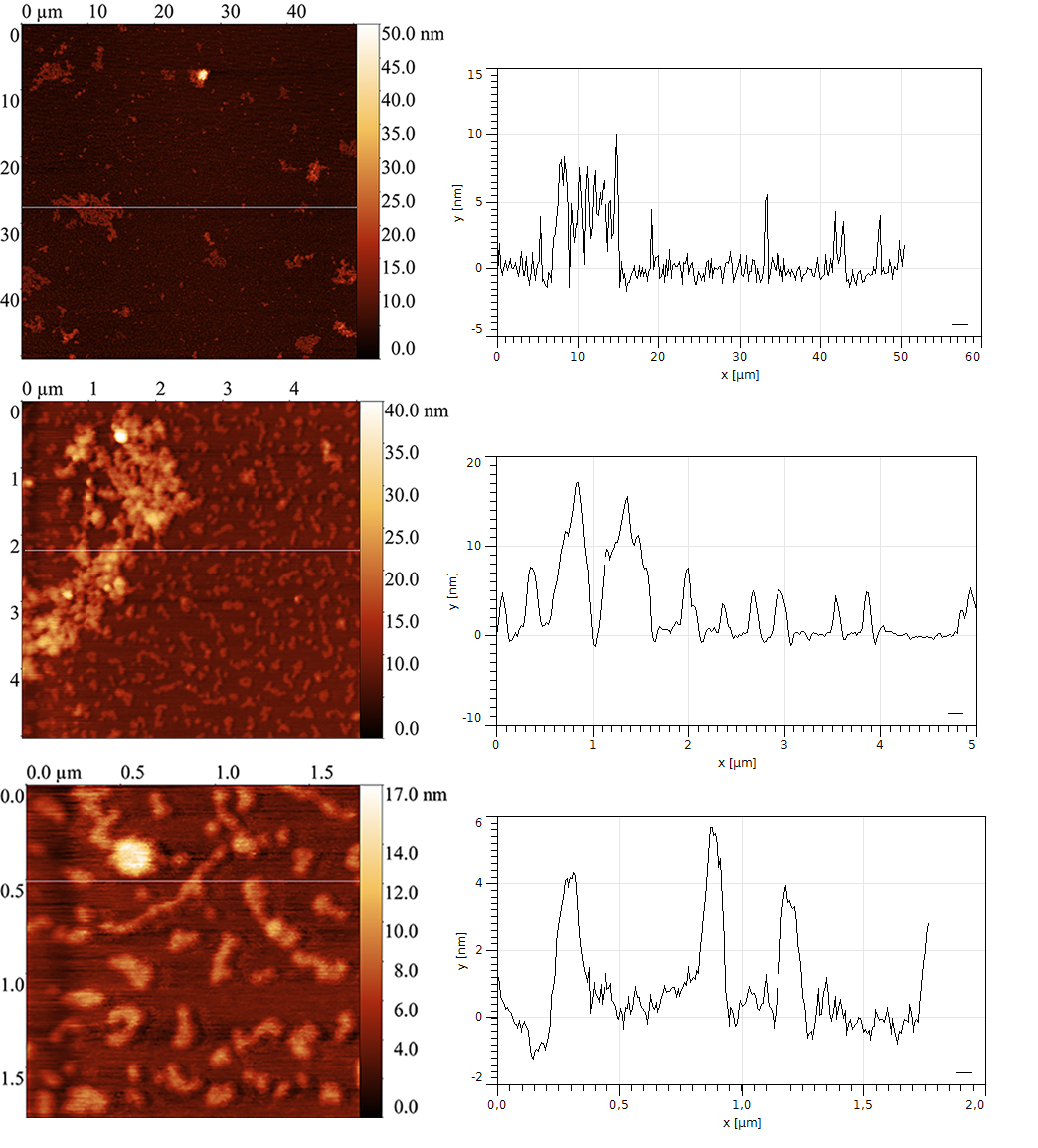


Supplementary Fig. S3. Atomic force microscopy of sMyBP-C with heights marked.

The original image is here:

<https://drive.google.com/drive/folders/1oyOQtXYT475O34OxCol1TXhBkNg_k3fS?usp=sharing>

**Skeletal myosin binding protein-C forms a special type of nontoxic amyloid aggregates *in vitro***

*Liya Bobyleva^1^, Sergey Shumeyko^1^, Elmira Yakupova^1^, Alexey Surin^2,3,4^, Oxana Galzitskaya^1,2^, Hiroshi Kihara^5^, Alexander Timchenko^6^, Maria Timchenko^7^, Nikita Penkov^8^, Alexey Nikulin^9^, Mariya Suvorina^2^, Nikolay Molochkov^10^, Mikhail Lobanov^2^, Roman Fadeev^11^, Ivan Vikhlyantsev^1,^* & Alexander Bobylev^1,^**

^1^Laboratory of the Structure and Functions of Muscle Proteins, Institute of Theoretical and Experimental Biophysics, Russian Academy of Sciences, Pushchino, Moscow Region 142290, Russia; ^2^Laboratory of Bioinformatics and Proteomics, Institute of Protein Research, Russian Academy of Sciences, Pushchino, Moscow Region 142290, Russia; ^3^Biological Testing Laboratory, Branch of the Shemyakin–Ovchinnikov Institute of Bioorganic Chemistry, Russian Academy of Sciences, Pushchino, Moscow Region 142290, Russia; ^4^Laboratory of the Biochemistry of Pathogenic Microorganisms, State Research Centre for Applied Microbiology and Biotechnology, Obolensk, Serpukhov District, Moscow Region, 142279, Russia; ^5^Department of Early Childhood Education, Himeji-Hinomoto College, 890 Koro, Kodera-cho, Himeji 679-2151, Japan; ^6^Group of Experimental Research and Engineering of Oligomeric Structures, Institute of Protein Research, Russian Academy of Sciences, Pushchino, Moscow Region 142290, Russia; ^7^Laboratory of Applied Enzymology, FRC PSCBR, Russian Academy of Sciences, Pushchino, Moscow Region 142290, Russia; ^8^Laboratory of the Methods of Optical Spectral Analysis, Institute of Cell Biophysics, Russian Academy of Sciences, FRC PSCBR RAS, Pushchino, Moscow Region 142290, Russia; ^9^Laboratory for Structural Studies of the Translational Apparatus, Institute of Protein Research, Russian Academy of Sciences, Pushchino, Moscow Region 142290, Russia; ^10^Laboratory of NMR Investigations of Biosystems, Institute of Theoretical and Experimental Biophysics, Russian Academy of Sciences, Pushchino, Moscow Region 142290, Russia; ^11^Laboratory of Pharmacological Regulation of Cell Resistance, Institute of Theoretical and Experimental Biophysics, Russian Academy of Sciences, Pushchino, Moscow Region 142290, Russia.


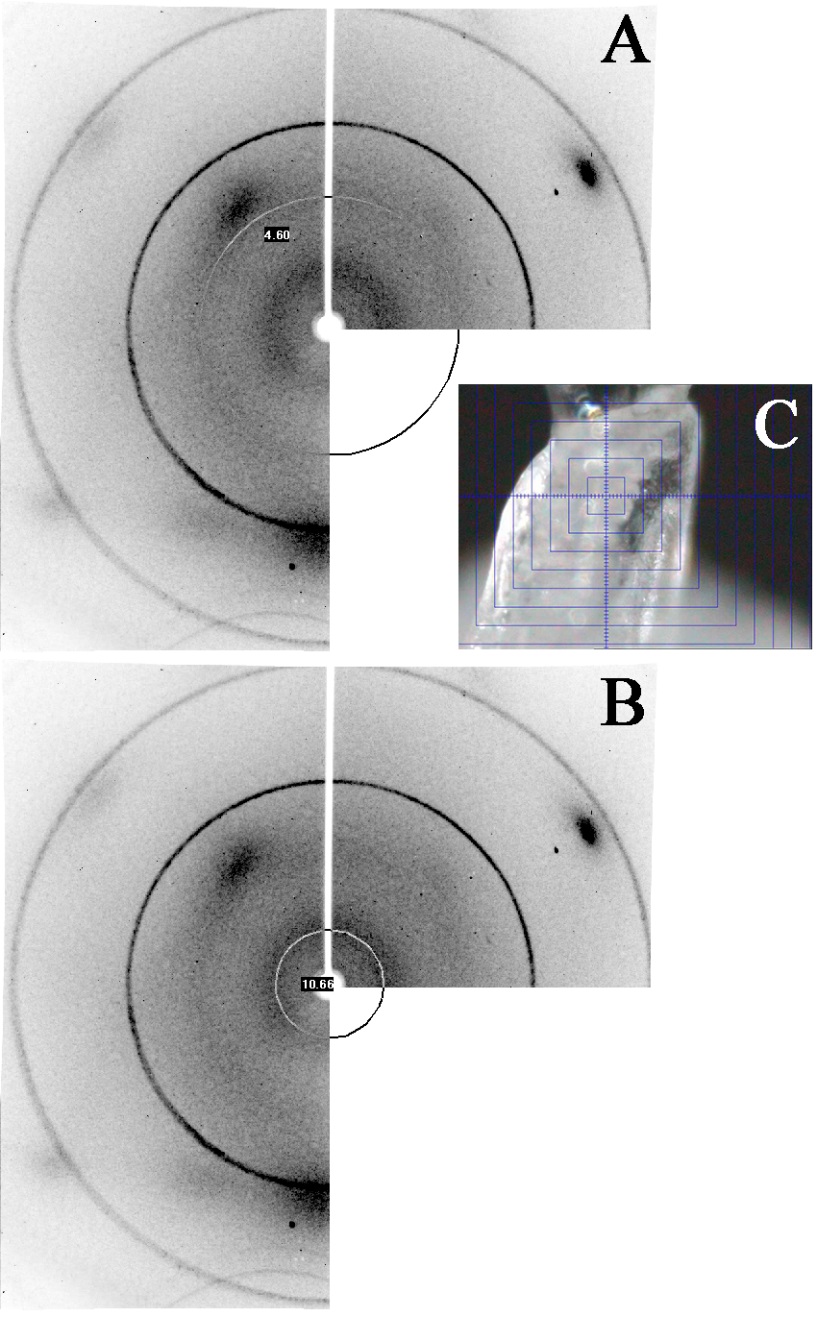


Supplementary Fig. S4. A,B, X-ray diffraction analysis revealed ring-shaped diffuse X-ray reflections of sMyBP-C aggregates. Calibration rings corresponding to distances of 10.66 Å and 4.60 Å are shown on top of the reflections. C, a crystal of aggregated sMyBP-C on the surface of a paraffin head, prepared according to the technique described in Materials and Methods (X-ray diffraction Section) of the manuscript. The X-ray beam pointing area is shown.

The original files are here:

<https://drive.google.com/drive/folders/1MSoMUzhFPHf0RR6xxMGeQZf3Q5OmDlN-?usp=sharing>

**Myosin binding protein-C forms amyloid-like aggregates *in vitro***

*Liya Bobyleva^1^, Sergey Shumeyko^1^, Elmira Yakupova^1^, Alexey Surin^2,3,4^, Oxana Galzitskaya^1,2^, Hiroshi Kihara^5^, Alexander Timchenko^6^, Maria Timchenko^7^, Nikita Penkov^8^, Alexey Nikulin^9^, Mariya Suvorina^2^, Nikolay Molochkov^10^, Mikhail Lobanov^2^, Roman Fadeev^11^, Ivan Vikhlyantsev^1,^* & Alexander Bobylev^1,^**

^1^Laboratory of the Structure and Functions of Muscle Proteins, Institute of Theoretical and Experimental Biophysics, Russian Academy of Sciences, Pushchino, Moscow Region 142290, Russia; ^2^Laboratory of Bioinformatics and Proteomics, Institute of Protein Research, Russian Academy of Sciences, Pushchino, Moscow Region 142290, Russia; ^3^Biological Testing Laboratory, Branch of the Shemyakin–Ovchinnikov Institute of Bioorganic Chemistry, Russian Academy of Sciences, Pushchino, Moscow Region 142290, Russia; ^4^Laboratory of the Biochemistry of Pathogenic Microorganisms, State Research Centre for Applied Microbiology and Biotechnology, Obolensk, Serpukhov District, Moscow Region, 142279, Russia; ^5^Department of Early Childhood Education, Himeji-Hinomoto College, 890 Koro, Kodera-cho, Himeji 679-2151, Japan; ^6^Group of Experimental Research and Engineering of Oligomeric Structures, Institute of Protein Research, Russian Academy of Sciences, Pushchino, Moscow Region 142290, Russia; ^7^Laboratory of Applied Enzymology, FRC PSCBR, Russian Academy of Sciences, Pushchino, Moscow Region 142290, Russia; ^8^Laboratory of the Methods of Optical Spectral Analysis, Institute of Cell Biophysics, Russian Academy of Sciences, FRC PSCBR RAS, Pushchino, Moscow Region 142290, Russia; ^9^Laboratory for Structural Studies of the Translational Apparatus, Institute of Protein Research, Russian Academy of Sciences, Pushchino, Moscow Region 142290, Russia; ^10^Laboratory of NMR Investigations of Biosystems, Institute of Theoretical and Experimental Biophysics, Russian Academy of Sciences, Pushchino, Moscow Region 142290, Russia; ^11^Laboratory of Pharmacological Regulation of Cell Resistance, Institute of Theoretical and Experimental Biophysics, Russian Academy of Sciences, Pushchino, Moscow Region 142290, Russia.

**Supplementary Table S3. Identity between different domains of ssMyBPC protein**

| name | fs | ls | n | Ig-like___1 | Ig-like___2 | Ig-like___3 | Ig-like___4 | Ig-like___5 | Ig-like___6 | Ig-like___7 |
| --- | --- | --- | --- | --- | --- | --- | --- | --- | --- | --- |
|  |  |  |  | 73 | 90 | 91 | 89 | 98 | 95 | 95 |
| Ig-like___1 | 72 | 144 | 73 |  | 4% | 10% | 0% | 0% | 0% | 7% |
| Ig-like___2 | 251 | 340 | 90 | 4% |  | 28% | 28% | 4% | 12% | 10% |
| Ig-like___3 | 341 | 431 | 91 | 10% | 28% |  | 28% | 0% | 15% | 16% |
| Ig-like___4 | 432 | 520 | 89 | 0% | 28% | 28% |  | 20% | 0% | 17% |
| Ig-like___5 | 522 | 619 | 98 | 0% | 4% | 0% | 20% |  | 24% | 27% |
| Ig-like___6 | 837 | 931 | 95 | 0% | 12% | 15% | 0% | 24% |  | 14% |
| Ig-like___7 | 1047 | 1141 | 95 | 7% | 10% | 16% | 17% | 27% | 14% |  |
|  |  |  |  |  |  |  |  |  |  |  |

| name | fs | ls | n | Fibro___1 | Fibro___2 | Fibro___3 |
| --- | --- | --- | --- | --- | --- | --- |
|  |  |  |  | 100 | 112 | 96 |
| Ig-like___1 | 72 | 144 | 73 | 0% | 0% | 0% |
| Ig-like___2 | 251 | 340 | 90 | 0% | 12% | 0% |
| Ig-like___3 | 341 | 431 | 91 | 2% | 0% | 7% |
| Ig-like___4 | 432 | 520 | 89 | 0% | 5% | 8% |
| Ig-like___5 | 522 | 619 | 98 | 3% | 0% | 0% |
| Ig-like___6 | 837 | 931 | 95 | 0% | 9% | 0% |
| Ig-like___7 | 1047 | 1141 | 95 | 5% | 3% | 7% |

| name | fs | ls | n | none___1 | none___2 |
| --- | --- | --- | --- | --- | --- |
|  |  |  |  | 71 | 106 |
| Ig-like___1 | 72 | 144 | 73 | 0% | 0% |
| Ig-like___2 | 251 | 340 | 90 | 0% | 0% |
| Ig-like___3 | 341 | 431 | 91 | 9% | 0% |
| Ig-like___4 | 432 | 520 | 89 | 3% | 0% |
| Ig-like___5 | 522 | 619 | 98 | 5% | 5% |
| Ig-like___6 | 837 | 931 | 95 | 0% | 0% |
| Ig-like___7 | 1047 | 1141 | 95 | 0% | 0% |

| name | fs | ls | n | Fibro___1 | Fibro___2 | Fibro___3 |
| --- | --- | --- | --- | --- | --- | --- |
|  |  |  |  | 100 | 112 | 96 |
| Fibro___1 | 622 | 721 | 100 |  | 30% | 24% |
| Fibro___2 | 722 | 833 | 112 | 30% |  | 21% |
| Fibro___3 | 934 | 1029 | 96 | 24% | 21% |  |

| name | fs | ls | n | none___1 | none___2 |
| --- | --- | --- | --- | --- | --- |
|  |  |  |  | 71 | 106 |
| Fibro___1 | 622 | 721 | 100 | 5% | 0% |
| Fibro___2 | 722 | 833 | 112 | 4% | 0% |
| Fibro___3 | 934 | 1029 | 96 | 16% | 0% |

| name | fs | ls | n | none___1 | none___2 |
| --- | --- | --- | --- | --- | --- |
|  |  |  |  | 71 | 106 |
| none___1 | 1 | 71 | 71 |  | 0% |
| none___2 | 145 | 250 | 106 | 0% |  |

fs, the first residue in a domain

1s, the last residue in a domain

n, the number of amino acid residues in a domain

**Identity was calculated by the formula:**

*Id* = 2 ⋅ *N*_id_/ (*L*_1_ + *L*_2_)

*N*_id_, number of identical residues in an alignment

*L*, number of residues in a domain

ssMyBP-C domains were taken from <http://www.uniprot.org/uniprot/Q00872>

fsMyBP-C domains were taken from <http://www.uniprot.org/uniprot/Q14324>

For cases of over 50 residues between domains, a none pseudodomain was formed

FnIII-like = Fibronectin type-III

The original files are here:

<https://drive.google.com/drive/folders/11NOeq_xR8epSUuImYQhbUJbAel7TjlcM?usp=sharing>

**Myosin binding protein-C forms amyloid-like aggregates *in vitro***

*Liya Bobyleva^1^, Sergey Shumeyko^1^, Elmira Yakupova^1^, Alexey Surin^2,3,4^, Oxana Galzitskaya^1,2^, Hiroshi Kihara^5^, Alexander Timchenko^6^, Maria Timchenko^7^, Nikita Penkov^8^, Alexey Nikulin^9^, Mariya Suvorina^2^, Nikolay Molochkov^10^, Mikhail Lobanov^2^, Roman Fadeev^11^, Ivan Vikhlyantsev^1,^* & Alexander Bobylev^1,^**

^1^Laboratory of the Structure and Functions of Muscle Proteins, Institute of Theoretical and Experimental Biophysics, Russian Academy of Sciences, Pushchino, Moscow Region 142290, Russia; ^2^Laboratory of Bioinformatics and Proteomics, Institute of Protein Research, Russian Academy of Sciences, Pushchino, Moscow Region 142290, Russia; ^3^Biological Testing Laboratory, Branch of the Shemyakin–Ovchinnikov Institute of Bioorganic Chemistry, Russian Academy of Sciences, Pushchino, Moscow Region 142290, Russia; ^4^Laboratory of the Biochemistry of Pathogenic Microorganisms, State Research Centre for Applied Microbiology and Biotechnology, Obolensk, Serpukhov District, Moscow Region, 142279, Russia; ^5^Department of Early Childhood Education, Himeji-Hinomoto College, 890 Koro, Kodera-cho, Himeji 679-2151, Japan; ^6^Group of Experimental Research and Engineering of Oligomeric Structures, Institute of Protein Research, Russian Academy of Sciences, Pushchino, Moscow Region 142290, Russia; ^7^Laboratory of Applied Enzymology, FRC PSCBR, Russian Academy of Sciences, Pushchino, Moscow Region 142290, Russia; ^8^Laboratory of the Methods of Optical Spectral Analysis, Institute of Cell Biophysics, Russian Academy of Sciences, FRC PSCBR RAS, Pushchino, Moscow Region 142290, Russia; ^9^Laboratory for Structural Studies of the Translational Apparatus, Institute of Protein Research, Russian Academy of Sciences, Pushchino, Moscow Region 142290, Russia; ^10^Laboratory of NMR Investigations of Biosystems, Institute of Theoretical and Experimental Biophysics, Russian Academy of Sciences, Pushchino, Moscow Region 142290, Russia; ^11^Laboratory of Pharmacological Regulation of Cell Resistance, Institute of Theoretical and Experimental Biophysics, Russian Academy of Sciences, Pushchino, Moscow Region 142290, Russia.

**Supplementary Table S4. Identity between different domains of fsMyBP-C protein**

| name | fs | ls | n | Ig-like___1 | Ig-like___2 | Ig-like___3 | Ig-like___4 | Ig-like___5 | Ig-like___6 | Ig-like___7 |
| --- | --- | --- | --- | --- | --- | --- | --- | --- | --- | --- |
|  |  |  |  | 104 | 90 | 93 | 101 | 100 | 95 | 94 |
| Ig-like___1 | 50 | 153 | 104 |  | 24% | 14% | 0% | 10% | 22% | 10% |
| Ig-like___2 | 255 | 344 | 90 | 24% |  | 26% | 19% | 15% | 0% | 12% |
| Ig-like___3 | 345 | 437 | 93 | 14% | 26% |  | 23% | 22% | 0% | 14% |
| Ig-like___4 | 438 | 538 | 101 | 0% | 19% | 23% |  | 8% | 0% | 22% |
| Ig-like___5 | 539 | 638 | 100 | 10% | 15% | 22% | 8% |  | 25% | 24% |
| Ig-like___6 | 838 | 932 | 95 | 22% | 0% | 0% | 0% | 25% |  | 7% |
| Ig-like___7 | 1048 | 1141 | 94 | 10% | 12% | 14% | 22% | 24% | 7% |  |

| name | fs | ls | n | Fibro___1 | Fibro___2 | Fibro___3 |
| --- | --- | --- | --- | --- | --- | --- |
|  |  |  |  | 97 | 96 | 96 |
| Ig-like___1 | 50 | 153 | 104 | 0% | 0% | 0% |
| Ig-like___2 | 255 | 344 | 90 | 0% | 3% | 13% |
| Ig-like___3 | 345 | 437 | 93 | 5% | 0% | 0% |
| Ig-like___4 | 438 | 538 | 101 | 0% | 9% | 0% |
| Ig-like___5 | 539 | 638 | 100 | 7% | 0% | 0% |
| Ig-like___6 | 838 | 932 | 95 | 0% | 0% | 5% |
| Ig-like___7 | 1048 | 1141 | 94 | 2% | 0% | 0% |

| name | fs | ls | n | none___1 | none___2 |
| --- | --- | --- | --- | --- | --- |
|  |  |  |  | 49 | 101 |
| Ig-like___1 | 50 | 153 | 104 | 14% | 0% |
| Ig-like___2 | 255 | 344 | 90 | 0% | 0% |
| Ig-like___3 | 345 | 437 | 93 | 0% | 5% |
| Ig-like___4 | 438 | 538 | 101 | 5% | 0% |
| Ig-like___5 | 539 | 638 | 100 | 3% | 13% |
| Ig-like___6 | 838 | 932 | 95 | 0% | 0% |
| Ig-like___7 | 1048 | 1141 | 94 | 3% | 0% |

| name | fs | ls | n | Fibro___1 | Fibro___2 | Fibro___3 |
| --- | --- | --- | --- | --- | --- | --- |
|  |  |  |  | 97 | 96 | 96 |
| Fibro___1 | 641 | 737 | 97 |  | 26% | 25% |
| Fibro___2 | 739 | 834 | 96 | 26% |  | 26% |
| Fibro___3 | 935 | 1030 | 96 | 25% | 26% |  |

| name | fs | ls | n | none___1 | none___2 |
| --- | --- | --- | --- | --- | --- |
|  |  |  |  | 49 | 101 |
| Fibro___1 | 641 | 737 | 97 | 0% | 7% |
| Fibro___2 | 739 | 834 | 96 | 3% | 0% |
| Fibro___3 | 935 | 1030 | 96 | 0% | 6% |

| name | fs | ls | n | none___1 | none___2 |
| --- | --- | --- | --- | --- | --- |
|  |  |  |  | 49 | 101 |
| none___1 | 1 | 49 | 49 |  | 8% |
| none___2 | 154 | 254 | 101 | 8% |  |

fs, the first residue in a domain

1s, the last residue in a domain

n, the number of amino acid residues in a domain

**Identity was calculated by the formula:**

*Id* = 2 ⋅ *N*_id_/ (*L*_1_ + *L*_2_)

*N*_id_, number of identical residues in an alignment

*L*, number of residues in a domain

ssMyBP-C domains were taken from <http://www.uniprot.org/uniprot/Q00872>

fsMyBP-C domains were taken from <http://www.uniprot.org/uniprot/Q14324>

For cases of over 50 residues between domains, a none pseudodomain was formed

FnIII-like = Fibronectin type-III

The original files are here:

<https://drive.google.com/drive/folders/11NOeq_xR8epSUuImYQhbUJbAel7TjlcM?usp=sharing>
