# Supplementary figures and images for: Myosin Binding Protein-C Forms Amyloid-Like Aggregates In Vitro
Source: Int J Mol Sci. 2021 Jan 13;22(2):731. doi: 10.3390/ijms22020731 (PMC7828380; doi:10.3390/ijms22020731)

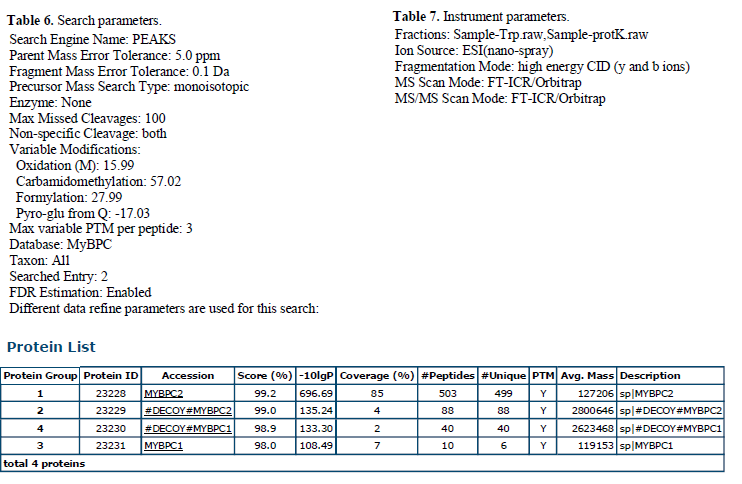

Supplement: Supplementary file 1 [file ijms-22-00731-s001.zip › ijms-1057004 SI-done/Supplementing Figures/S1.bmp]

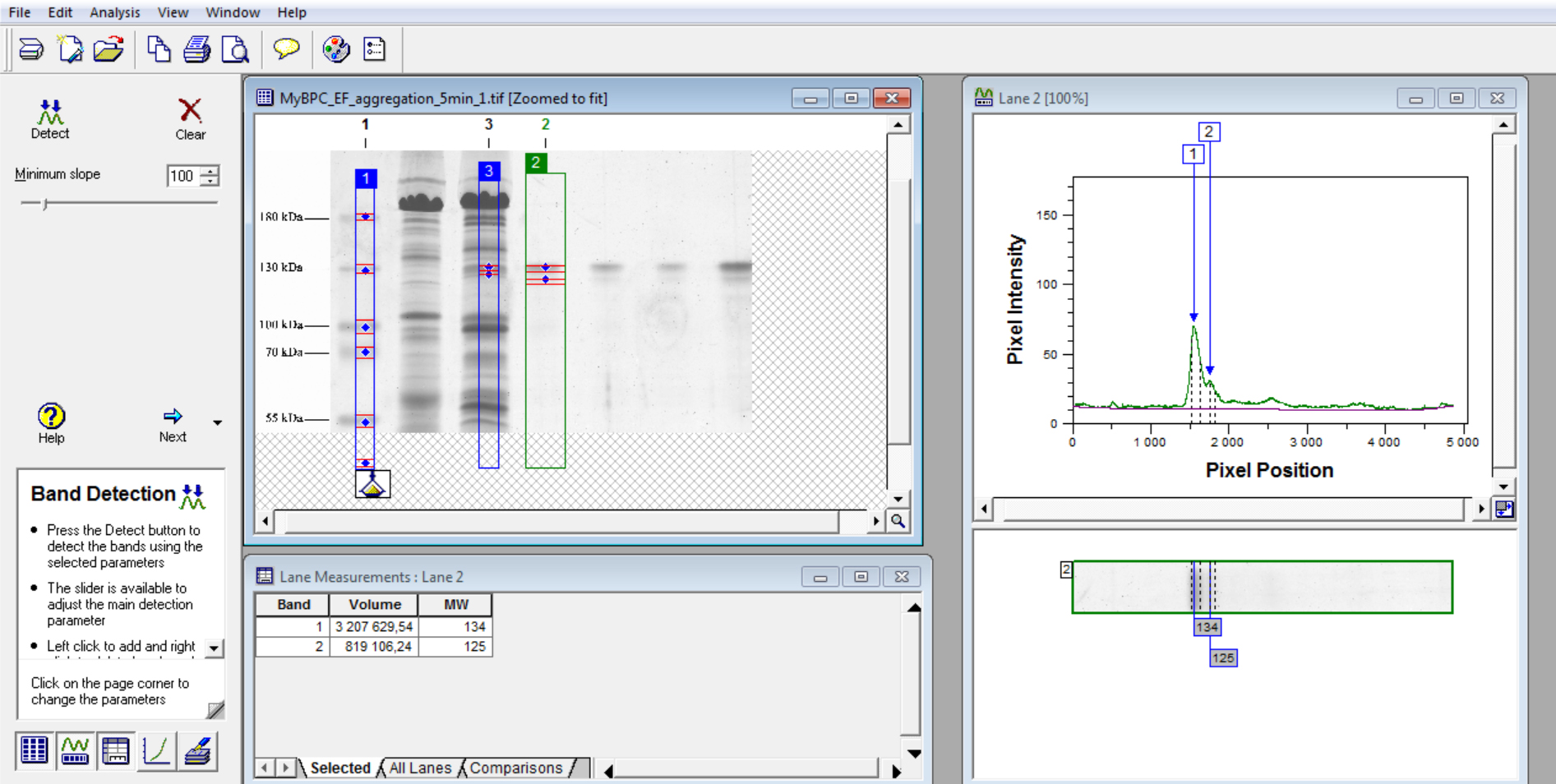

Supplement: Supplementary file 1 [file ijms-22-00731-s001.zip › ijms-1057004 SI-done/Supplementing Figures/S2.jpg]

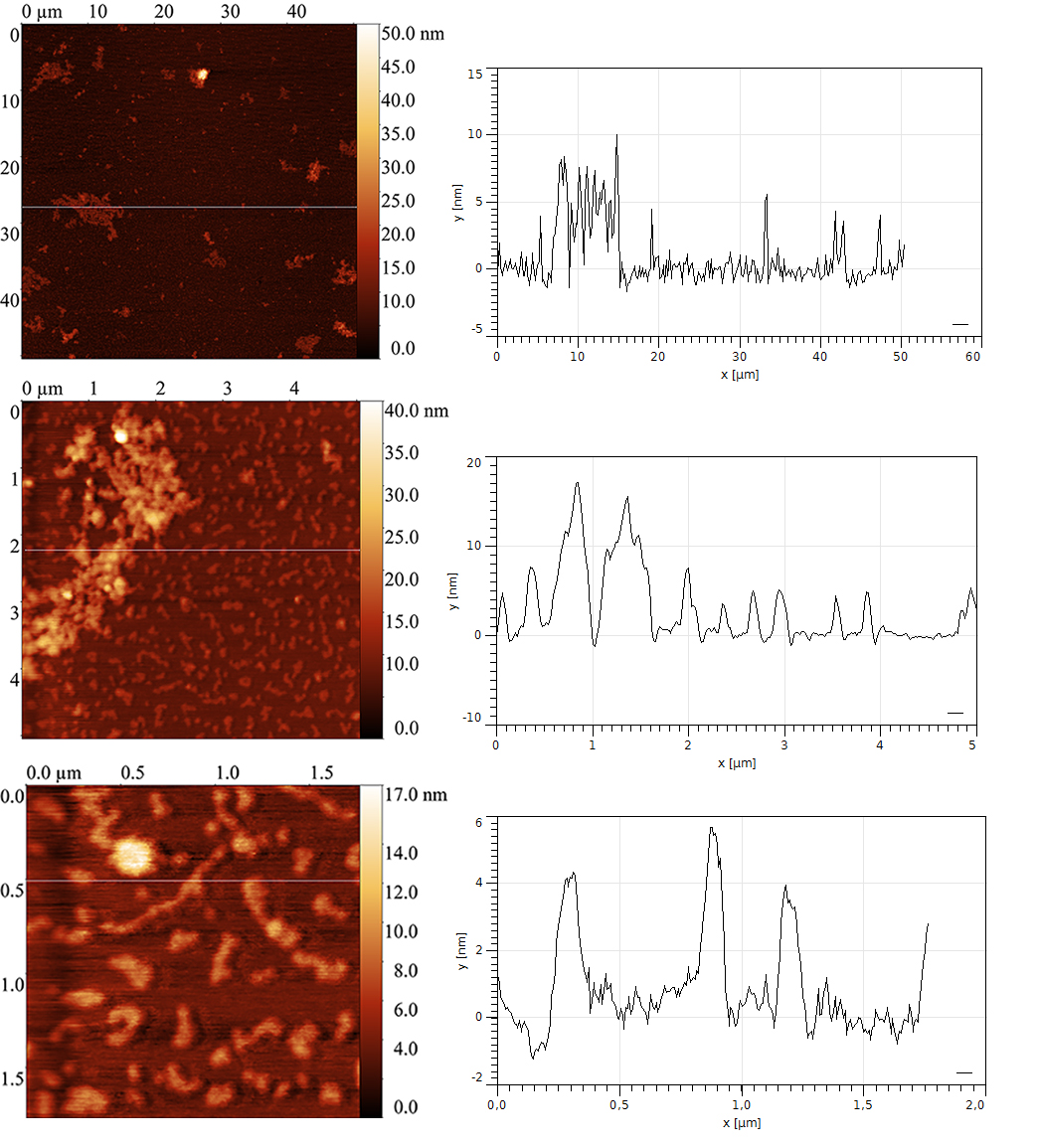

Supplement: Supplementary file 1 [file ijms-22-00731-s001.zip › ijms-1057004 SI-done/Supplementing Figures/S4.jpg]

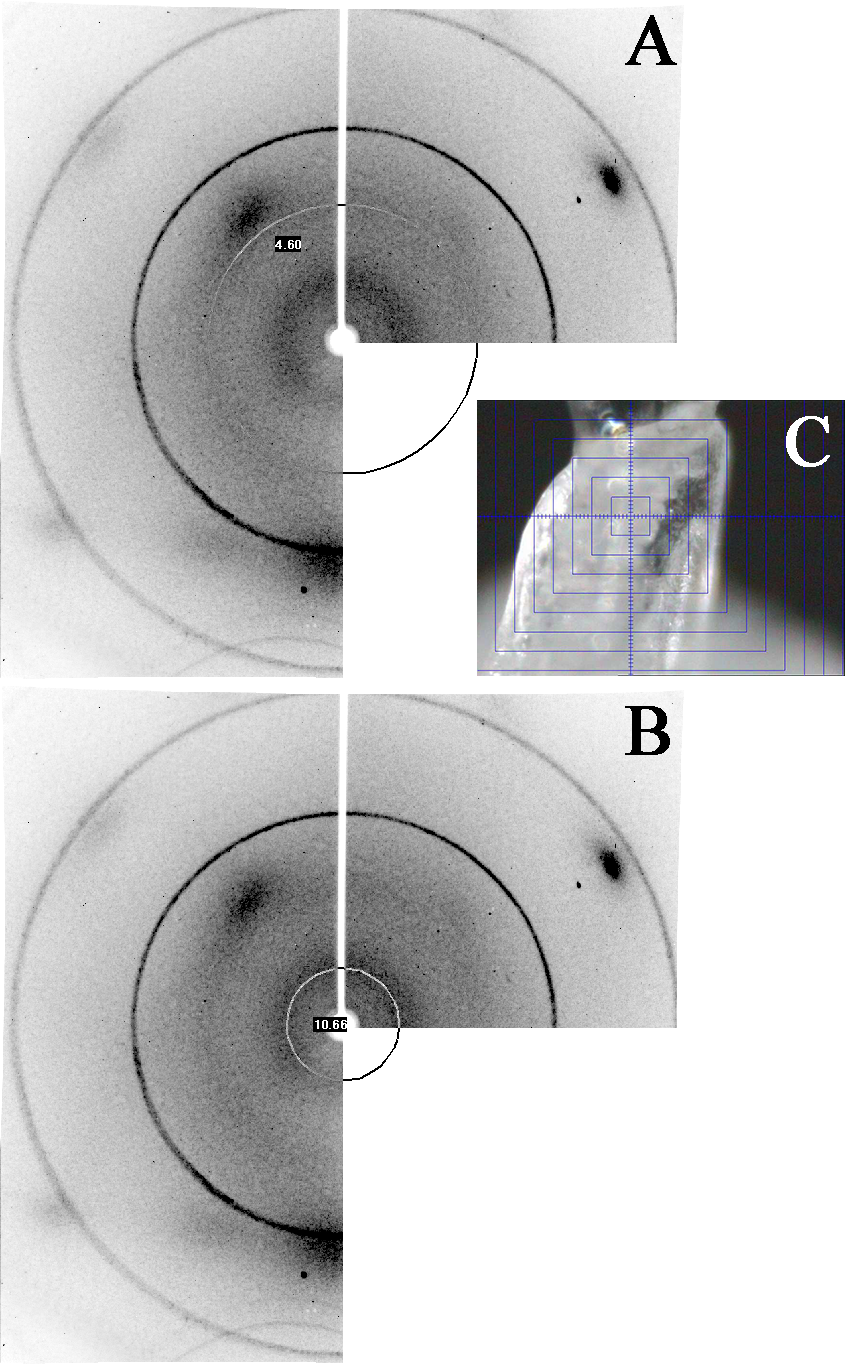

Supplement: Supplementary file 1 [file ijms-22-00731-s001.zip › ijms-1057004 SI-done/Supplementing Figures/S5.png]
